# Histamine Derived from Probiotic *Lactobacillus reuteri* Suppresses TNF via Modulation of PKA and ERK Signaling

**DOI:** 10.1371/journal.pone.0031951

**Published:** 2012-02-22

**Authors:** Carissa M. Thomas, Teresa Hong, Jan Peter van Pijkeren, Peera Hemarajata, Dan V. Trinh, Weidong Hu, Robert A. Britton, Markus Kalkum, James Versalovic

**Affiliations:** 1 Interdepartmental Program in Cell and Molecular Biology, Baylor College of Medicine, Houston, Texas, United States of America; 2 Department of Immunology, Beckman Research Institute of the City of Hope, Duarte, California, United States of America; 3 Department of Microbiology and Molecular Genetics, Michigan State University, East Lansing, Michigan, United States of America; 4 Department of Molecular Virology and Microbiology, Baylor College of Medicine, Houston, Texas, United States of America; 5 Department of Pathology and Immunology, Baylor College of Medicine, Houston, Texas, United States of America; 6 Department of Pathology, Texas Children's Hospital, Houston, Texas, United States of America; Charité, Campus Benjamin Franklin, Germany

## Abstract

Beneficial microbes and probiotic species, such as *Lactobacillus reuteri*, produce biologically active compounds that can modulate host mucosal immunity. Previously, immunomodulatory factors secreted by *L. reuteri* ATCC PTA 6475 were unknown. A combined metabolomics and bacterial genetics strategy was utilized to identify small compound(s) produced by *L. reuteri* that were TNF-inhibitory. Hydrophilic interaction liquid chromatography-high performance liquid chromatography (HILIC-HPLC) separation isolated TNF-inhibitory compounds, and HILIC-HPLC fraction composition was determined by NMR and mass spectrometry analyses. Histamine was identified and quantified in TNF-inhibitory HILIC-HPLC fractions. Histamine is produced from L-histidine via histidine decarboxylase by some fermentative bacteria including lactobacilli. Targeted mutagenesis of each gene present in the histidine decarboxylase gene cluster in *L. reuteri* 6475 demonstrated the involvement of histidine decarboxylase pyruvoyl type A (*hdcA*), histidine/histamine antiporter (*hdcP*), and *hdcB* in production of the TNF-inhibitory factor. The mechanism of TNF inhibition by *L. reuteri*-derived histamine was investigated using Toll-like receptor 2 (TLR2)-activated human monocytoid cells. Bacterial histamine suppressed TNF production via activation of the H_2_ receptor. Histamine from *L. reuteri* 6475 stimulated increased levels of cAMP, which inhibited downstream MEK/ERK MAPK signaling via protein kinase A (PKA) and resulted in suppression of TNF production by transcriptional regulation. In summary, a component of the gut microbiome, *L. reuteri*, is able to convert a dietary component, L-histidine, into an immunoregulatory signal, histamine, which suppresses pro-inflammatory TNF production. The identification of bacterial bioactive metabolites and their corresponding mechanisms of action with respect to immunomodulation may lead to improved anti-inflammatory strategies for chronic immune-mediated diseases.

## Introduction

Microbes that colonize the gastrointestinal (GI) tract produce diverse extracellular signals such as p40/p75 from *Lactobacillus rhamnosus* GG and AI-3 from commensal *E. coli*. A majority of the low-molecular weight molecules produced by beneficial microbes have not been characterized biochemically, but these compounds modulate specific host immune and physiologic responses [1], [2]. *Lactobacillus reuteri* is a beneficial microbe that is indigenous to the GI tract of diverse mammalian species including humans, and at least two different strains are considered probiotics [3], [4]. *L. reuteri* ATCC PTA 6475 (strain 6475) confers multiple potential benefits to the human host including the production of antimicrobial compounds [5], [6], biosynthesis of B complex vitamins [7], and the secretion of immunomodulatory factors [8], [9].

Relatively few probiotic-derived compounds with immunomodulatory properties have been identified. Known strain-specific probiotic immunomodulins include lactic acid, the surface layer A protein of *L. acidophilus* NCFM, peptidoglycan-derived muropeptides, and capric acid [10]–[13]. TNF-inhibitory molecules or immunomodulins produced by *L. reuteri* 6475 were previously unknown. Because probiotic species, including *L. reuteri*, can target specific signaling pathways and immune responses, these bacterial strains may represent future therapeutic agents that could serve to suppress chronic inflammation. Limited mechanistic studies are a current barrier to targeted application of probiotics for specific immune-mediated human diseases, despite the fact that numerous probiotics have been linked to effects on specific intracellular signaling pathways in different human and murine cell types [14]. The central limitation is the lack of well-defined molecular signals that are produced by beneficial microbes, and the discovery of these microbial metabolites will enable the development of new drugs and probiotics.

Probiotics may affect the disease course of patients with chronic immune-mediated diseases such as pouchitis or necrotizing enterocolitis via modulation of the immune system [15]–[17]. For example, select probiotic strains suppress secretion of Th1, Th2 or Th17 cytokines by various immune cells, which may correspondingly diminish patterns of mucosal inflammation in the GI tract [18], [19]. *L. reuteri* 6475 inhibits production of TNF, a pro-inflammatory cytokine, from monocyte-derived macrophages isolated from children with Crohn's disease as well as TLR2- and TLR4-activated human and murine monocytoid cell lines [8], [18]. Similar to other probiotics [14], *L. reuteri* 6475 suppresses activation of the AP-1 transcription factor, which regulates the expression of pro-inflammatory cytokine genes in response to activation of Toll-like receptors [8]. The effects of *L. reuteri* strains and other probiotic species on signaling pathways between cell surface receptors and mitogen-activated protein kinase (MAPK)-regulated transcription factors, such as AP-1, are unknown and deserve further exploration.

Engagement of TLRs by microbial-derived signals results in the activation of the MAPK pathways, extracellular signal-regulated kinase (ERK), c-Jun N-terminal kinase (JNK), and p38. Activation of ERK or JNK results in translocation of AP-1 and transcription of pro-inflammatory genes [20]. ERK signaling affects TNF production [21]. TLR stimulation results in Ras/c-Raf-dependent activation of the MEK/ERK pathway [22]. This Ras/c-Raf-dependent activation of ERK can be modulated by the cAMP/protein kinase A (PKA) signaling cascade induced by engagement of G protein-coupled receptors (GPCR), and defects in GPCR signaling may result in chronic colitis [23]–[25]. In the intestine, destructive inflammation driven by TNF requires activation of MAPKs, ERK and p38 [26]. These MAPK signaling pathways are potential targets for modulation by beneficial microbes, resulting in suppression of TNF and inflammation.

In this study, we employed a combination of metabolomics and bacterial genetics approaches to identify potential immunomodulins (TNF-inhibitory factors) produced by *L. reuteri* 6475. TNF-inhibitory compounds were isolated by HILIC-HPLC and identified by NMR and mass spectrometry. The biogenic amine, histamine was identified and quantified in TNF-inhibitory HILIC-HPLC fractions. *L. reuteri* 6475 possesses three genes potentially involved in histamine production from the essential amino acid L-histidine - histidine decarboxylase pyruvoyl type A (*hdcA*), histidine/histamine antiporter (*hdcP*), and *hdcB*. Gene inactivation demonstrated that each gene contributes to the TNF-inhibitory phenotype of strain 6475. Mechanistic studies in human monocytoid cells revealed that *L. reuteri*-derived histamine potently inhibited TNF production via signaling through the histamine H_2_ receptor, a GPCR that is linked to downstream PKA activity and MAPK signaling.

## Results

### *Lactobacillus reuteri* produces histamine

Compounds with TNF inhibitory activity were isolated from *L. reuteri* cell pellets treated with trifluoroacetic acid (TFA) acidified water and supernatants from liquid cultures of *L. reuteri* 6475. Components of the aqueous TFA-treated cell pellets were separated based on relative hydrophobicity using HILIC-HPLC. The fractions were tested for retention of TNF-inhibitory compounds by activating human monocytoid cells (THP-1) with a TLR2 agonist in the presence of individual HILIC-HPLC fractions and monitoring TNF levels by quantitative ELISA. *L. reuteri* 6475 grown in a defined medium with glucose (LDMIIIG) as the sole carbon source produced TNF-inhibitory factors that were retained in three separate HILIC-HPLC fractions, B3, B5 and B6 (Figure S1A). The B4 fraction consistently lacked TNF-inhibitory activity.

**Figure 1:**
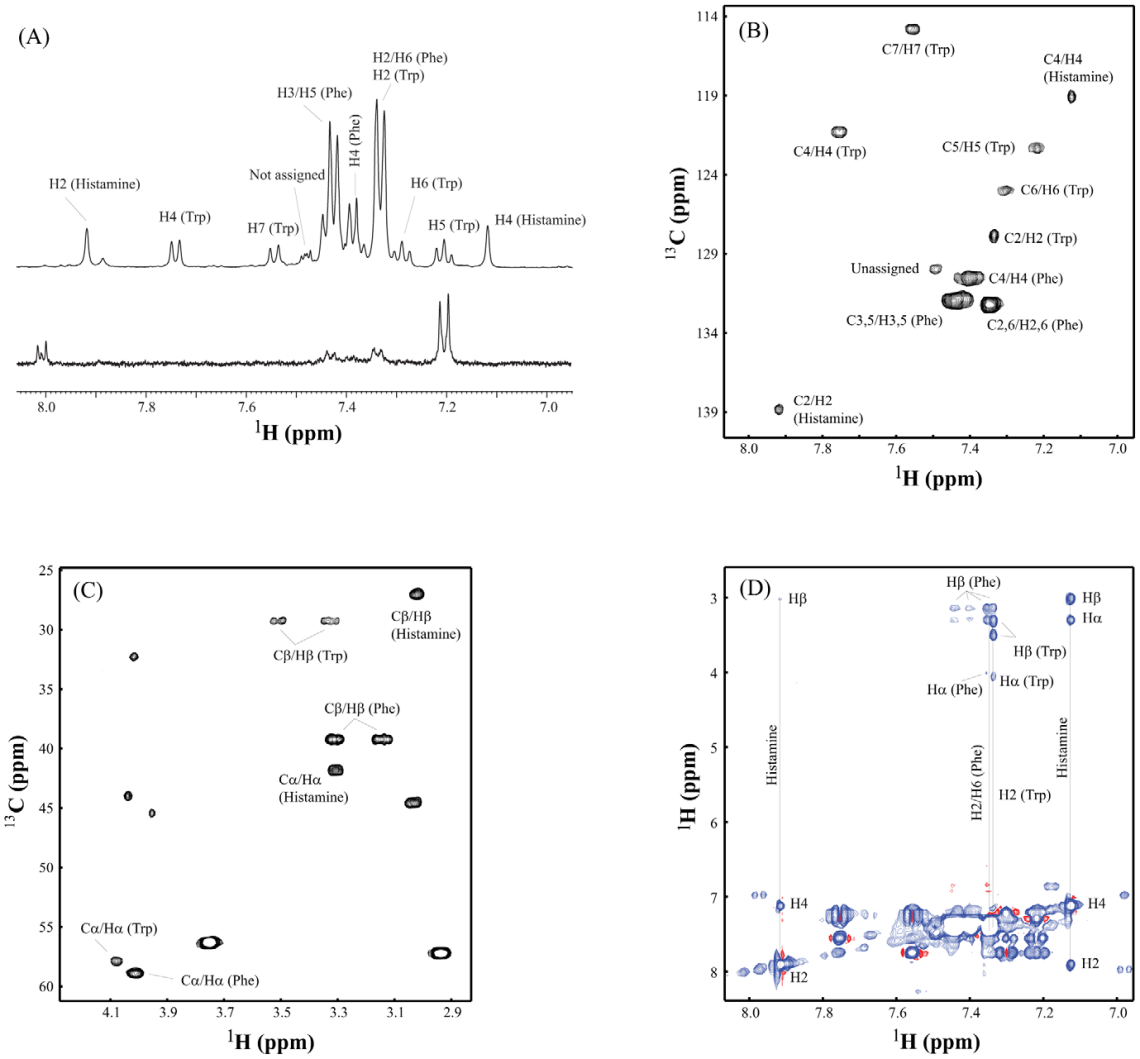
Identification of histamine in TNF-inhibitory fractions of *L. reuteri* 6475 by NMR. ^1^H NMR analysis demonstrated differences in the composition of HILIC-HPLC fractions from *L. reuteri* 6475. **A.** Top and bottom spectra show the 1D ^1^H NMR of TNF-inhibitory fraction B3 and fraction B4, respectively. The assigned peaks of phenylalanine (Phe), tryptophan (Trp), and histamine from fraction B3 are labeled on the top spectrum. Two complementary 2D NMR techniques were used to identify compounds unique to TNF-inhibitory fraction B3. **B.** Aromatic peaks of ^1^H-^13^C-HSQC from Phe, Trp, and histamine. **C.** Aliphatic peaks of ^1^H-^13^C-HSQC from Phe, Trp, and histamine. **D.** The TOCSY spectrum further identified the spin system of Phe, Trp, and histamine unique to TNF-inhibitory fraction B3.

The TNF-inhibitory HILIC-HPLC fraction B3 was analyzed by one-dimensional (1D) ^1^H NMR and compared to the neighboring non-TNF-inhibitory fraction B4. A unique series of peaks with a chemical shift between 7.0–8.0 ppm, which indicate the presence of aromatic or heterocyclic compounds, were observed in fraction B3 (Figure 1A, top spectrum) but not in fraction B4 (Figure 1A, bottom spectrum). To identify the compounds yielding these peaks, two-dimensional (2D) ^1^H-^13^C-heteronuclear single quantum coherence (HSQC) data were acquired with fraction B3, and compounds were identified using MetaboMiner software [27]. The compounds present in fraction B3 were tryptophan (Trp), phenylalanine (Phe), histamine, and one unknown peak (Figure 1B and 1C). All observable ^1^H-^13^C cross peaks of these three compounds are labeled in Figure 1B and 1C, which were identical to those compounds listed in the database of MetaboMiner. Compound identification was further confirmed using an additional 2D NMR method, total correlation spectroscopy (TOCSY), as shown in Figure 1D. Tryptophan and phenylalanine were components of the defined bacterial growth medium. Histidine, but not histamine, was part of the bacterial growth medium, suggesting that histidine may be converted to histamine by *L. reuteri*.

**Figure 2:**
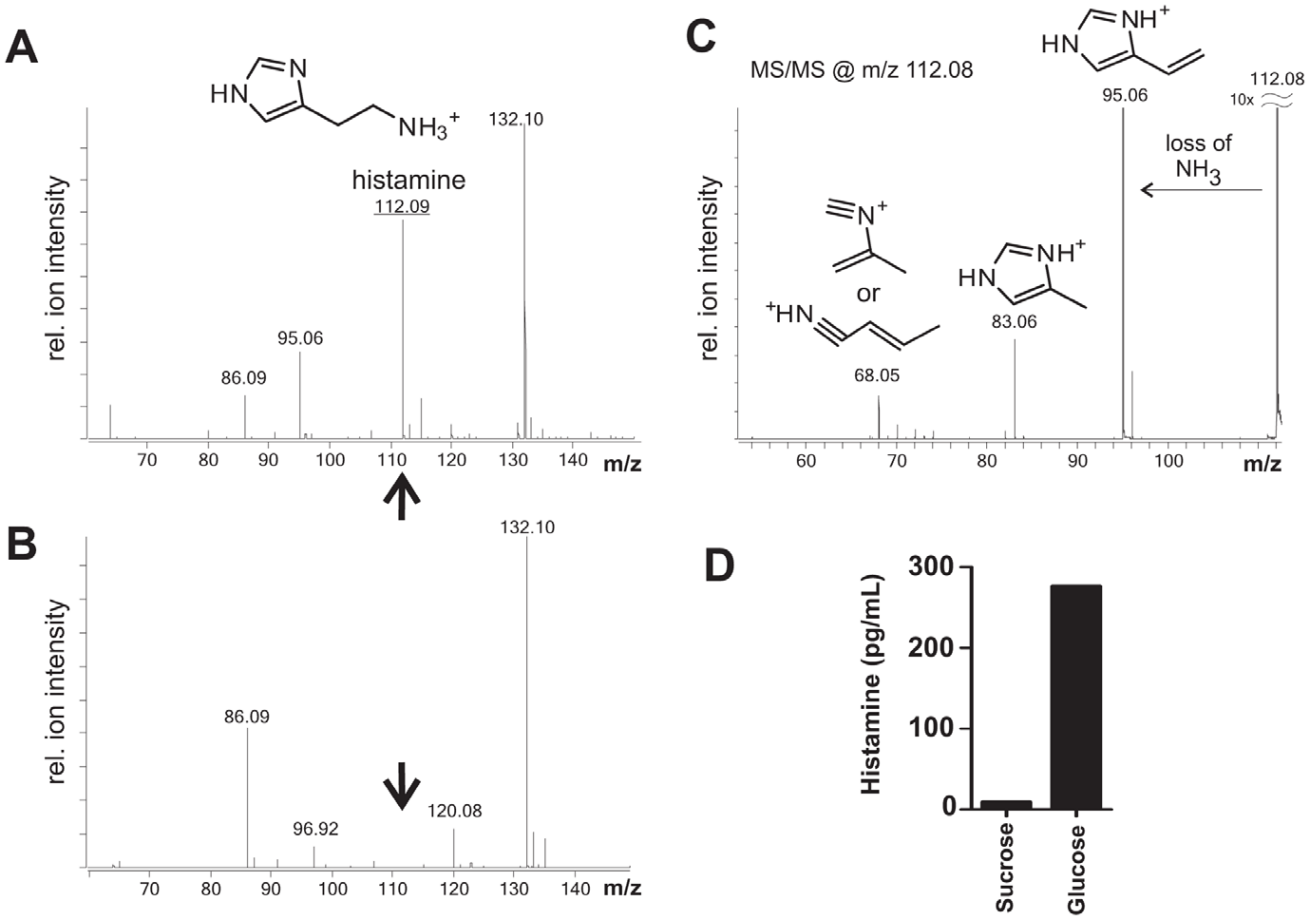
The production of histamine by *L. reuteri* 6475 was confirmed by mass spectrometry. Examination of TNF-inhibitory HILIC-HPLC fractions from *L. reuteri* 6475 grown in LDMIIIG confirmed the presence of histamine by ESI qTOF MS. **A.** ESI qTOF MS of TNF-inhibitory HILIC-HPLC fraction B3 from strain 6475 grown in glucose. Histamine was detected at m/z 112.08. **B.** ESI qTOF MS of non-TNF inhibitory HILIC-HPLC fraction B3 from strain 6475 grown in sucrose. Histamine was not detected at m/z 112.08. **C.** MS/MS analysis of m/z 112.08 confirmed that the compound is histamine. **D.** *L. reuteri* culture supernatants from sucrose or glucose-containing media were analyzed for histamine concentration by triple quadrupole mass spectrometry. Histamine (pg/mL) was detected only in culture supernatants from glucose-containing medium cultures.

The presence of histamine in the aqueous TFA cell pellet extracts was confirmed by electrospray time-of-flight mass spectrometry (ESI qTOF MS). Analysis of fraction B3 from strain 6475 grown in LDMIIIG revealed histamine detected at m/z of 112.09 (Figure 2A). The TNF-inhibitory phenotype of strain 6475 can be altered by different growth conditions. For example, strain 6475 grown in a sucrose-based medium (LDMIIIS) did not produce identifiable TNF inhibitory factors and demonstrated a diminished TNF-inhibitory phenotype. Strain 6475 cultivated in LDMIIIS served as a negative control for these studies (Figure S1B). Fraction B3 from strain 6475 grown in LDMIIIS did not contain histamine at m/z of 112.09 (Figure 2B). Histamine was confirmed as the unique compound in fraction B3 from 6475 grown in LDMIIIG via MS/MS analysis of m/z 112.08 (Figure 2C). Finally, histamine was detected in bacterial cell-free supernatants from liquid cultures of *L. reuteri* 6475 grown in LDMIIIG, suggesting bacterial secretion of histamine (Figure 2D).

### Quantification of *Lactobacillus reuteri*-derived histamine by triple quadrupole mass spectrometry

**Figure 3:**
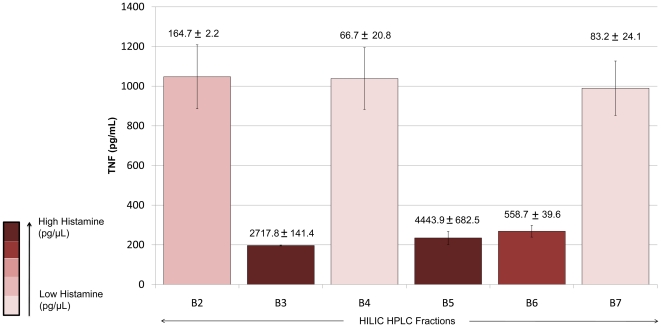
Quantification of histamine by triple quadrupole mass spectrometry. Histamine in HILIC-HPLC fractions B2–B7 from *L. reuteri* 6475 grown in a glucose-containing medium was quantified by triple quadrupole mass spectrometry. The y-axis indicates TNF production from activated human monocytoid cells. The concentration of histamine (pg/µL) is indicated above each bar graph. Elevated levels of histamine correlated with TNF inhibition.

Triple quadrupole mass spectrometry is an established method of quantifying small molecular compounds [28]. This method was used to quantify histamine in a restricted set of HILIC-HPLC fractions from aqueous TFA-treated cell pellets of *L. reuteri* 6475 grown in LDMIIIG. The standard curve used to quantify histamine is shown in Figure S2. All TNF-inhibitory HILIC-HPLC fractions (B3, B5, and B6) contained elevated concentrations of histamine (>500 pg/µL), while non-TNF-inhibitory HILIC-HPLC fractions contained less than 200 pg/µL histamine (Figure 3). The “gap” in immunomodulatory activity at fraction B4 correlated with low histamine content when compared to the neighboring fractions B3, B5, and B6. It is not entirely clear why histamine eluted in two peaks from the HILIC-HPLC column. The supernatant from liquid cultures of *L. reuteri* 6475 grown in LDMIIIG contained measurable quantities of histamine (approximately 300 pg/mL), whereas the culture supernatant of the same strain grown in LDMIIIS had minimal amounts of histamine (Figure 2D).

### The histidine decarboxylase gene cluster contributes to the TNF-inhibitory phenotype of *L. reuteri* 6475

**Figure 4:**
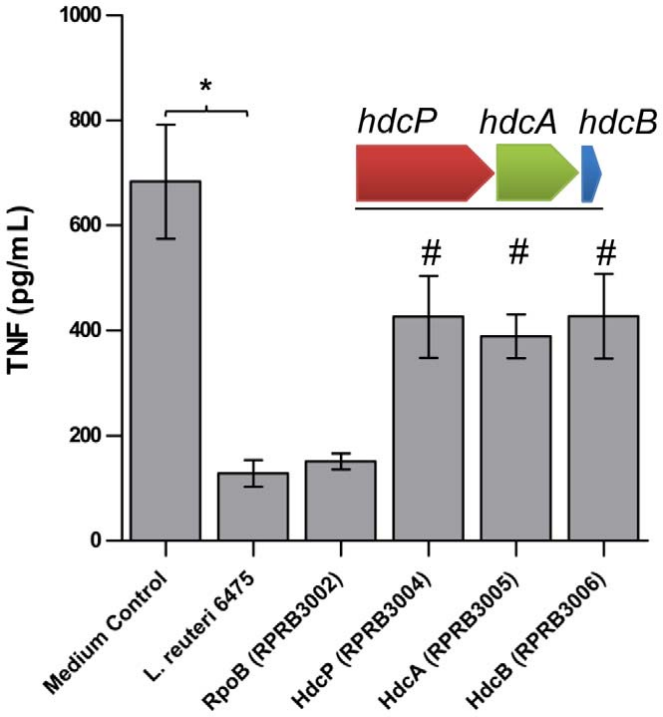
The histidine decarboxylase gene cluster contributed to the TNF-inhibitory phenotype of *L. reuteri* 6475. The histidine decarboxylase gene cluster consists of three genes, *hdcP* (RPRB3004), *hdcA* (RPRB3005), and *hdcB* (RPRB3006). The organization of the cluster is depicted above the bar graph. Inactivation by targeted mutagenesis in any one of these genes resulted in a partial loss (approximately 40%) of TNF suppression by *L. reuteri* 6475. Results represent the mean ± SD (n = 9), ^*^p value<0.05 compared to medium control, ^#^p value<0.05 compared to *L. reuteri* 6475.

Three genes, histidine/histamine antiporter (*hdcP*), histidine decarboxylase pyruvoyl type A (*hdcA*), and hdcB (*hdcB*) (thought to catalyze the maturation of HdcA [29]), are clustered together in the chromosome of *L. reuteri* 6475. The histidine decarboxylase gene cluster has been characterized in other *Lactobacillus* species [30], [31]. Relative expression of the histidine decarboxylase genes in wild-type 6475 was increased *in vitro* by supplementing the growth medium with histidine (Figure S3A). Transcriptomics studies comparing *L. reuteri* 6475 grown in LDMIIIS (lacking TNF inhibition) versus LDMIIIG (retained TNF inhibition) demonstrated significant down-regulation of all three genes in the presence of sucrose (*hdcP*: 5.5 fold down-regulated, *hdcA*: 30.1 fold down-regulated, *hdcB*: 11.8 fold down-regulated, p-value<0.05). Using RecT-dependent recombineering, we introduced in-frame stop codons in *hdcP* (locus tag HMPREF0536_1229), *hdcA* (locus tag HMPREF0536_1230), and *hdcB* (locus tag HMPREF0536_1231) [32], [33]. Inactivation of any one of the three genes in the histidine decarboxylase gene cluster was sufficient to cause a partial reduction (approximately 40%) of TNF-inhibition compared to the wild-type 6475 strain (Figure 4), suggesting that each gene contributes to the TNF-inhibitory phenotype of *L. reuteri* 6475. No effect on TNF inhibition was seen with inactivation of a control gene not involved in histamine production (in-frame stop codon in the RNA polymerase β subunit gene, *rpoB*; locus tag HMPREF0536_0828) (Figure 4). Histamine production was significantly reduced in the histidine decarboxylase mutants compared to wild-type 6475 despite supplementation with histidine in the growth medium (Figure S3B). A partial, rather than complete abrogation of activity in the *L. reuteri* 6475 histidine decarboxylase mutants suggests that other active TNF-inhibitory factors are being produced.

### Histamine inhibits TNF production via the H_2_ receptor

**Figure 5:**
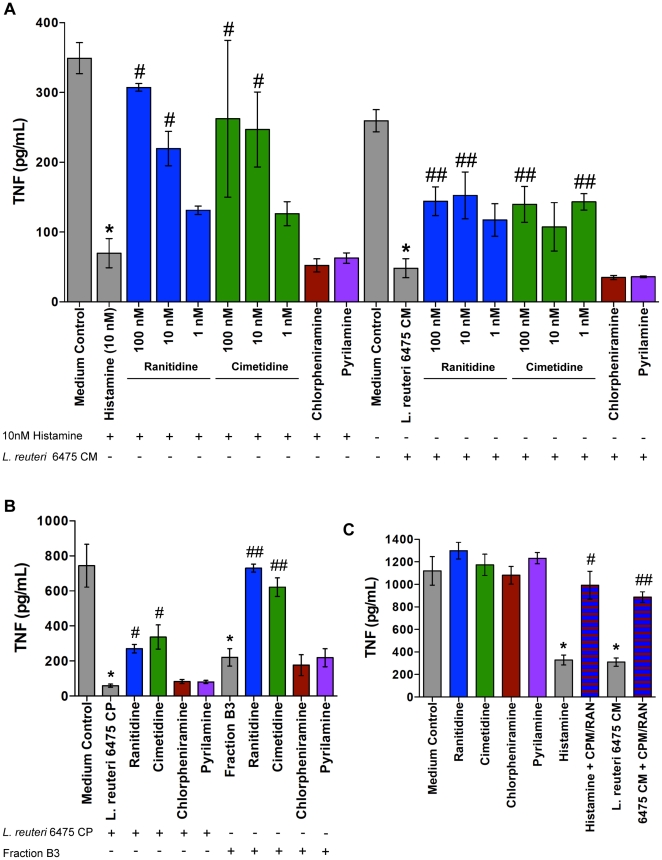
*L. reuteri*-derived histamine inhibited TNF production via the H_2_ receptor. **A.** Histamine inhibited TNF production, and suppression of TNF was blocked by specific H_2_ receptor antagonists in a concentration-dependent manner. *L. reuteri* 6475 conditioned media (CM) containing histamine inhibited TNF production, an effect partially blocked by specific H_2_R antagonists. The presence or absence of histamine (10 nM) or *L. reuteri* 6475 CM is indicated by a “+” or “−” respectively. Colored bars indicate treatment with select antagonists - ranitidine (H_2_R; blue), cimetidine (H_2_R; green), chlorpheniramine (H_1_R; red), and pyrilamine (H_1_R; purple). H_1_R antagonists were tested at 100 nM. Results represent the mean ± SD (n = 3), ^*^p value<0.05 compared to medium control, ^#^p value<0.05 compared to histamine, ^##^p value<0.05 compared to *L. reuteri* 6475 CM. **B.** TFA-treated cell pellets containing histamine suppressed TNF production, an effect partially blocked by specific H_2_R antagonists. HILIC-HPLC fraction B3, which was enriched for histamine, inhibited TNF production, an effect completely blocked by specific H_2_R antagonists. All antagonists were tested at 100 nM. The presence or absence of *L. reuteri* 6475 CP or Fraction B3 is indicated by a “+” or “−” respectively. Results represent the mean ± SD (n = 3), ^*^p value<0.05 compared to medium control, ^#^p value<0.05 compared to *L. reuteri* 6475 CP, ^##^p value<0.05 compared to fraction B3. **C.** Histamine receptor antagonists alone did not affect TNF production. All antagonists were tested at 100 nM. The H_1_R antagonist, chlorpheniramine (CPM), did not prevent the H_2_R antagonist, ranitidine (RAN), from blocking TNF suppression by histamine (10 nM) and *L. reuteri* 6475 CM. Blue bars with red stripes indicate combined treatment with CPM plus RAN. Results represent the mean ± SD (n = 3), ^*^p value<0.05 compared to medium control, ^#^p value<0.05 compared to histamine, ^##^p value<0.05 compared to *L. reuteri* 6475 CM.

Histamine significantly blocked TNF production (80% inhibition compared to medium control, p-value<0.05) by TLR2-activated THP-1 cells (Figure 5A). Histamine can activate four different GPCRs; H_1_, H_2_, H_3_, and H_4_ receptors. Myeloid cells in the gut express H_1_ and H_2_ receptors and, to a lesser extent, H_4_ receptors [34]. Previous studies demonstrated that histamine inhibits TNF production via the H_2_ receptor [35], and flow cytometry analysis with histamine receptor-specific antibodies confirmed that THP-1 cells express H_1_ and H_2_ receptors (Figure S4). Histamine receptor-specific antagonists were used to determine which receptor was mediating the effect of histamine on TNF production. H_2_ receptor-specific antagonists, ranitidine and cimetidine, suppressed TNF-inhibition by histamine in a concentration dependent manner (Figure 5A). H_1_ receptor-specific antagonists, chlorpheniramine and pyrilamine, had no effect on TNF-inhibition by histamine (Figure 5A). *L. reuteri* 6475 conditioned media (CM) containing histamine significantly inhibited TNF compared to the medium control (81%, p-value<0.05), and this effect was partially blocked by antagonists of the H_2_ receptor (44–46% TNF inhibition compared to medium control), but not H_1_ receptor antagonists (Figure 5A). *L. reuteri* 6475-derived, TFA-treated, unfractionated cell pellet (CP) containing histamine suppressed TNF production (92%, p-value<0.05) (Figure 5B). As seen with strain 6475 CM, H_2_ receptor antagonists partially blocked (55–64% TNF inhibition compared to medium control) the effect of extracellular, cell surface-derived components of strain 6475 (Figure 5B), suggesting that multiple immunomodulins may be present in the bacterial supernatant and unfractionated cell surface preparation. The effect of TNF-inhibitory HILIC-HPLC fraction B3 (70% inhibition compared to medium control), which is enriched for histamine, was markedly abrogated by addition of H_2_ receptor antagonists (2–17% TNF inhibition compared to medium control) (Figure 5B). Histamine receptor antagonists alone did not affect TNF production by TLR2-activated THP-1 cells (Figure 5C). In addition, H_1_ receptor antagonists did not prevent H_2_ receptor antagonists from blocking the TNF-suppressive activity of histamine and *L. reuteri* 6475 CM (Figure 5C).

### Histamine increased intracellular cAMP and triggered PKA signaling that yielded TNF-inhibitory activity

**Figure 6:**
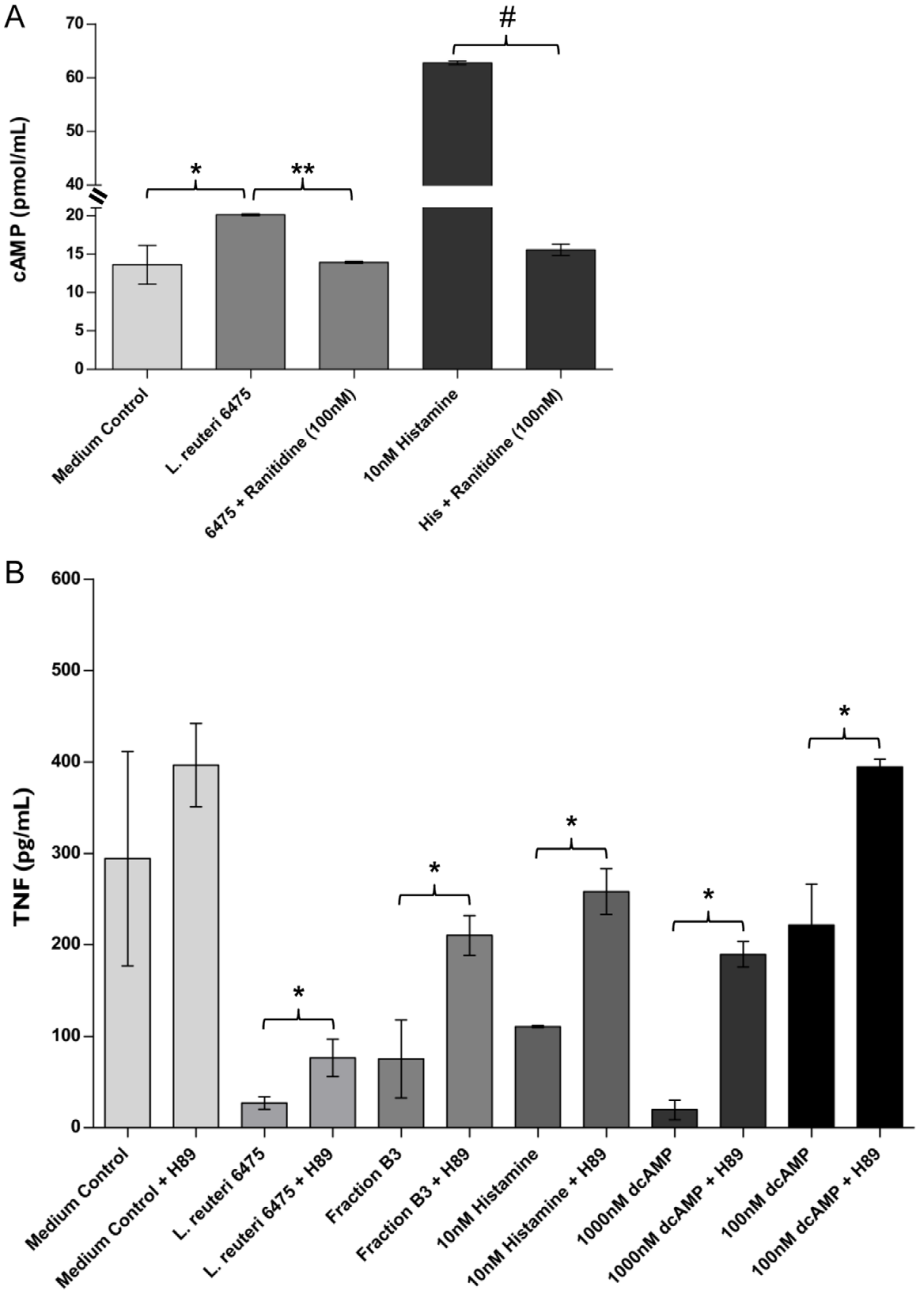
Stimulation of the H_2_ receptor increased cAMP and PKA activity was required for TNF suppression. Signaling pathway studies were performed to determine the effect of histamine on cAMP production and the role of PKA activity in TNF suppression. **A.** Treatment of activated THP-1 cells with *L. reuteri* 6475 CM or histamine increased intracellular cAMP. The increase in cAMP was blocked by a specific H_2_ receptor antagonist, ranitidine. Results represent the mean ± SD (n = 2), ^*^p value<0.05 compared to medium control, ^**^p value<0.05 compared to *L. reuteri* 6475, ^#^p value<0.05 compared to histamine. **B.** Inhibition of PKA activity partially blocked TNF suppression by TNF-inhibitory compounds *L. reuteri* 6475 CM, HILIC-HPLC fraction B3, histamine, and dcAMP. Results represent the mean ± SD (n = 9), ^*^p value<0.05 compared to H89 treated samples.

The H_2_ receptor is a GPCR that can activate adenylate cyclase (AC) and increase intracellular cAMP [36]. TNF can be inhibited at the level of transcription by cAMP and cAMP analogs [37]. Therefore, we wanted to determine if stimulating the H_2_ receptor in THP-1 cells with *L. reuteri*-derived histamine would increase intracellular cAMP and if increased amounts of cAMP would be sufficient to inhibit TNF production. THP-1 cells were stimulated with a TLR2 agonist in the presence of medium control, *L. reuteri* 6475 CM or histamine. These experiments were conducted with or without an H_2_ receptor antagonist (ranitidine), and intracellular levels of cAMP were measured by ELISA. Addition of *L. reuteri* 6475 CM yielded significantly increased quantities of cAMP in human monocytoid cells (Figure 6A), whereas treatment with ranitidine blocked the cAMP-stimulatory effect by *L. reuteri*. A similar inhibitory effect of ranitidine was observed with respect to the ability of histamine to elevate cAMP concentrations (Figure 6A). To determine if elevated cAMP could suppress TNF, a synthetic analog of cAMP, dibutyryl cAMP (dcAMP), was added to TLR2-stimulated THP-1 cells at varying concentrations. The addition of 10–1000 nM dcAMP significantly inhibited TNF production (Figure S5). Stimulation of the histamine H_2_ receptor resulted in increased cAMP, which blocked downstream TNF production in activated monocytoid cells.

The signal transduction cascade from cAMP to PKA has been linked to inhibition of the ERK MAPK signaling pathway. PKA can inhibit c-Raf activity, an upstream MAP3K to MEK1/2, which activates ERK [23], [24]. To determine if PKA activity was important for TNF suppression by *L. reuteri*-derived histamine, activated THP-1 cells were treated with a specific chemical inhibitor of PKA, H89, in the presence of either *L. reuteri* 6475 CM, HILIC-HPLC fraction B3, histamine or varying concentrations of dcAMP. The addition of H89, which inhibited PKA activity, partially blocked TNF inhibition by all of the TNF-inhibitory compounds (Figure 6B), demonstrating that PKA activity was important for suppression of TNF by histamine and dcAMP.

### Histamine signaling through the H_2_ receptor blocks activation of MEK1/2 and ERK1/2 and downstream TNF production

**Figure 7:**
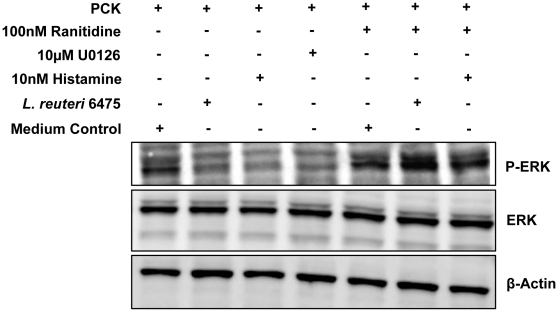
Stimulation of the H_2_ receptor inhibited the ERK signaling pathway. Immunoblot studies were performed to determine the effect of histamine on activation of the ERK signaling pathway. ERK was activated by phosphorylation on Thr202/Tyr204 (P-ERK). Treatment of TLR-stimulated THP-1 cells with *L. reuteri* 6475, histamine, or U0126 suppressed activation of ERK1/2, an effect that was significantly blocked in the presence of ranitidine. The β-actin antibody served as the protein loading control.

Previous studies demonstrated that PKA inhibits Ras/c-Raf activation of MEK/ERK MAPK signaling [23], [24]. Therefore, the phosphorylation states of MEK1/2 and ERK1/2 were examined by immunoblot to determine if histamine was, in fact, suppressing activation of the MEK/ERK signaling pathway. Treatment of activated THP-1 cells with *L. reuteri* 6475 CM, histamine or U0126, a specific chemical inhibitor of MEK activity which served as the positive control, blocked activation (phosphorylation) of both MEK1/2 and downstream ERK1/2 compared to the medium control (Figure 7 and Figure S6). Treatment with ranitidine partially restored activation of MEK1/2 and completely restored activation of ERK1/2 (Figure 7 and Figure S6), suggesting that inhibition of the MEK/ERK MAPK pathway occurred via H_2_ receptor activation. MEK1/2 and ERK1/2 protein quantities did not differ following these treatments. In summary, histamine derived from *L. reuteri* 6475 inhibited activation of MEK and downstream ERK, resulting in diminished TNF production by TLR2-stimulated monocytoid cells.

Engagement of TLRs results in activation of MAPK signaling cascades and downstream transcription factors such as AP-1 [20]. ERK is activated by the upstream MAP2K, MEK. To determine the role of ERK in TLR2-stimulated TNF production, THP-1 cells were treated with a specific MEK inhibitor, U0126, for varying amounts of time prior to stimulation with a TLR2 agonist, and the effect on human TNF production was monitored. Treatment with U0126 for a minimum of 30 min was sufficient to prevent TNF production (Figure S7). ERK1/2 is activated following TLR2 stimulation of THP-1 cells and is essential for stimulating TNF production in our model system.

## Discussion

**Figure 8:**
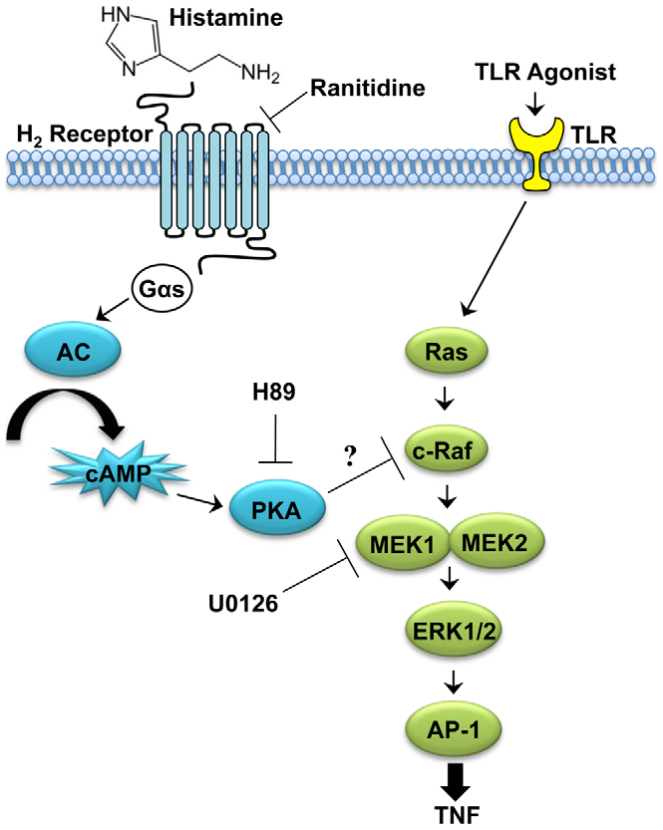
Pathway diagram of the mechanism of histamine inhibition of ERK activation. THP-1 cells activated with a TLR2 agonist activated the MEK/ERK MAPK signaling cascade to induce production of TNF. Histamine produced by *L. reuteri* 6475 engaged the H_2_ receptor on activated THP-1 cells. This G protein-coupled receptor activated adenylate cyclase (AC) to increase intracellular cAMP, which subsequently activated protein kinase A (PKA). PKA inhibited MEK/ERK activation and suppressed production of TNF. It was predicted that PKA inhibited MEK/ERK activation by inhibiting the activity of c-Raf. Specific H_2_ receptor antagonists, such as ranitidine, prevented histamine from activating the H_2_ receptor and blocked TNF suppression by histamine. H89, a specific inhibitor of PKA, prevented TNF suppression by histamine, demonstrating that PKA activity was necessary for histamine's effect on TNF. Blocking MEK activity with U0126 suppressed activation of ERK and downstream TNF production, demonstrating that ERK activation was necessary for production of TNF.

This report highlights the identification of the biogenic amine, histamine, as an immunoregulatory factor linking diet, the microbiome (*L. reuteri* strain 6475), and the innate immune system. For the first time, histamine is described as a probiotic immunomodulin that potentially explains the suppression of innate immunity by intestinal bacteria. Mechanistic studies demonstrated that *L. reuteri*-derived histamine inhibited human TNF production via signaling through the H_2_ receptor. Signaling through the H_2_ receptor increased intracellular cAMP, and PKA activity was necessary for TNF suppression by histamine. Finally, histamine blocked activation of the MEK/ERK MAPK signaling pathway and presumably suppressed transcription of the TNF gene by inhibition of AP-1 translocation to the nucleus (Figure 8). It has been demonstrated previously that *L. reuteri* 6475 suppresses transcription of TNF by inhibiting activation of c-Jun and AP-1 [8].

Histamine is well known for its pro-inflammatory effects in allergy and anaphylaxis; however several studies have demonstrated anti-inflammatory or immunoregulatory functions of histamine. *In vitro* studies have shown that histamine can inhibit production of pro-inflammatory cytokines, IL-1, IL-12, and TNF from LPS-stimulated human monocytes and macrophages and this effect is reversed by H_2_ receptor antagonists [35], [38], [39]. Additionally, histamine can stimulate production of the anti-inflammatory cytokine, IL-10, via the H_2_ receptor [39]. Signaling through the H_2_ receptor results in decreased expression of the CD14 receptor, a receptor involved in LPS recognition, on the surface of human monocytes. This potential mechanism may be one explanation for the suppression of TNF production by histamine [40]. *In vivo* studies have also revealed an anti-inflammatory role for histamine. Treatment with dimaprit, a specific H_2_ receptor agonist, reduced plasma TNF levels in mouse models of endotoxin shock (LPS challenge) and hepatitis (LPS plus galactosamine challenge) [41]. Histamine was protective in a LPS-induced liver injury mouse model, and these effects were attenuated in a H_2_ receptor knock-out mouse [42]. In the gut, histamine may help protect against bacterial infection. Signaling through the H_2_ receptor in Peyer's patches helps prevent infection by *Yersinia enterocolitica* [43].

How does histamine exert both a pro- and anti-inflammatory effect on host cells? One possibility is that a structural modification distinguishes histamine as a pro- or anti-inflammatory compound. However, the fragmentation pattern of histamine in our MS/MS analysis revealed that histamine produced by *L. reuteri* is not covalently modified (Figure 2C). Another possible hypothesis is that the pro- or anti-inflammatory effect of histamine is concentration dependent. However, this possibility seems unlikely in the host since histamine is rapidly degraded by two intestinal enzymes, histamine-N-methyl transferase and diamine oxidase [44]. A more intriguing hypothesis for histamine's anti-inflammatory property is that the effect of histamine is determined by the expression and activation of histamine receptors on the target host cell [45]. In T lymphocytes, for example, the effect of histamine appears to be dependent on the class of histamine receptor that is activated. By signaling through the H_1_ receptor, histamine enhances Th1-type responses but suppresses both Th1 and Th2 responses via the H_2_ receptor [46]. Many cell types in the human GI tract express multiple histamine receptors. For example, immune cells including macrophages, mainly express H_1_ and H_2_ receptors with relatively low quantities of the H_4_ receptor, while intestinal epithelial cells (IECs) express both H_1_ and H_2_ receptors [34]. The distribution of histamine receptors in the GI tract indicates that the gut microbiota can signal to IECs and underlying immune cells. Locally produced histamine from *L. reuteri* may be activating H_2_ receptors on IECs as well as immune cells to suppress host mucosal immunity via inhibition of pro-inflammatory cytokines.

Blocking the H_2_ receptor with specific antagonists may exacerbate the disease course of some chronic inflammatory conditions since activation of the H_2_ receptor is responsible for the anti-inflammatory activity of histamine. H_2_ receptor antagonist treatment was associated with an increased incidence of necrotizing enterocolitis (NEC) in very low birth weight infants [47]. Guillet *et al*. proposed that decreased gastric acid and changes in pH caused by H_2_ antagonist treatment may contribute to NEC; however, the data presented here suggests an alternative pathogenic mechanism of inhibiting histamine's immunosuppressive properties via blockade of H_2_ receptors [47]. In the future, it will be important to evaluate closely the effects of histamine receptor antagonists in certain disease processes, such as NEC and inflammatory bowel disease (IBD), given our growing knowledge of the anti-inflammatory effects of *L. reuteri*-derived histamine.

Histamine is produced by decarboxylation of histidine, an essential amino acid for humans that is present in many dietary foods [48]. Histamine production by *L. reuteri* 6475 is increased by supplementing the bacterial growth medium with histidine. Supplementing the diet with certain amino acids, such as histidine, may modulate intestinal inflammation associated with IBD [49]. *In vitro* studies demonstrated that histidine inhibited production of the pro-inflammatory cytokine IL-8 from TNF-stimulated IECs [50]. Additionally, the effects of histidine were examined *in vivo* using an IL-10-deficient (IL-10^−/−^) cell transfer colitis model, and histidine exerted a protective effect against colitis [51]. Histidine is converted to histamine by the gut microbiota [52], and this bioconversion may be one explanation for the anti-inflammatory effects of histidine. The conversion from dietary histidine to histamine by *L. reuteri* could take place in the gut lumen in close proximity to cells, such as IECs and immune cells expressing the H_2_ receptor, resulting in an anti-inflammatory effect. Mice deficient in murine histidine decarboxylase activity demonstrate increased quantities of pro-inflammatory cytokines in intestinal tissue and augmented infiltration of activated myeloid cells in tissues [53].

Histidine supplementation increased expression of histidine decarboxylase genes and production of histamine by *L. reuteri* (Figure S3). In addition, histamine production can be modified by carbon sources present in the environment or growth media in the laboratory. Interestingly, these studies demonstrate that histamine production by *L. reuteri* is dependent on the presence of glucose in the growth media. *L. reuteri* loses the ability to inhibit TNF and produce histamine when grown in a sucrose-based medium: however, the TNF-inhibitory phenotype is retained if lactobacilli are grown in a glucose/fructose-based medium (Figure S8). The specific links between carbon sources and histamine production are currently unknown, but such knowledge will facilitate an improved understanding of diet-microbiome-host connections. Based on the results of these *in vitro* studies, examining the impact of diet on the production of histamine *in vivo* will be important for enhancing immunomodulation by *L. reuteri*.

Analysis of the Human Microbiome Project (HMP) and the reference genomes of gastrointestinal bacteria [54] for histidine decarboxylase gene homologs revealed that out of 349 reference genomes, only four genomes (all *Lactobacillus*) contained the complete histidine decarboxylase gene cluster (*L. reuteri* JCM 112, *L. reuteri* MM2-3, *L. reuteri* 6475, and *L. vaginalis* ATCC 49540 (Figure S9)). Additionally, histamine production appears to be a host-specific trait as only *L. reuteri* strains isolated from human hosts possess the histidine decarboxylase gene [55]. The limited distribution of the histidine decarboxylase gene cluster in the human gut microbiome suggests that *L. reuteri* has evolved to occupy a unique niche within its human host.

Probiotics produce active enzymes during their transit through the GI tract, and this production can be modified by diet. For example, *Streptococcus thermophilus* produced active β-galactosidase *in vivo*, and production was increased with dietary supplementation of lactose [56]. Transcriptomics studies of the complex gut microbiome are in their infancy. However, *in vivo* gene expression studies of probiotic strains such as *L. reuteri* and *L. plantarum* have been performed using *in vivo* expression technology (IVET). Genes induced *in vivo* were primarily involved in nutrient acquisition, stress response, and extracellular protein production [57], [58]. Mixed-species genomic microarray analysis of infant fecal samples demonstrated transcription of bifidobacterial genes involved in carbohydrate metabolism and exopolysaccharide and folate production *in vivo* [59]. The *in vivo* relative activities of bacterial promoters present in gut bacteria, including the promoter for the histidine biosynthetic pathway, were demonstrated by measuring luciferase activity in germ-free mice monoassociated with *Lactococcus lactis*. The activity of the histidine biosynthetic pathway promoter was dependent upon the concentration of histidine, indicating that histidine can regulate bacterial gene expression *in vivo* [60], [61]. The relative expression of genes in the histidine decarboxylase gene cluster by *L. reuteri in vivo* and the effects of histidine concentration are currently unknown.

Production of histamine by certain bacterial strains has caused alarm as a health risk in food and as a marker of food spoilage. For example, scombroid poisoning, a form of non-infectious foodborne disease, is due to production of highly elevated quantities of histamine and other biogenic amines by bacteria in spoiled food, particularly fish [62]. Fermented foods and beverages naturally contain small quantities of histamine due to the conversion of L-histidine by bacteria in food substances prior to ingestion, and histamine-producing strains have been excluded from foods due to secondary concerns about allergic reactions. The results of the current studies support the possible importance of dietary amino acids like histidine in foods, and subsequent biological conversion to active metabolites in the intestine at the site(s) of action. The localization of bacteria in the intestine and the relative distribution of histamine receptors in the intestinal mucosa will determine the efficacy and potency of immunomodulation by the microbiome. These studies do not advocate for oral ingestion of histamine, but rather for luminal conversion of dietary components to bacterial metabolites. Combining an essential amino acid, histidine, with indigenous bacteria like *L. reuteri* results in conversion to histamine at the site of activity and modulation of the host immune response in the gut.

We hypothesize that *L. reuteri* 6475 produces multiple immunomodulatory factors, including but not limited to histamine. Additional bioactive compounds may have co-eluted with histamine in the TNF-inhibitory HILIC-HPLC fractions B5 and B6. One-dimensional NMR identified several unique peaks that were not histamine in fractions B5 and B6 compared to neighboring non-TNF-inhibitory fractions. The identification and characterization of these unique peaks is an ongoing effort. The presence of multiple immunomodulins is supported by the observation that the histidine decarboxylase mutants did not completely lose the ability to suppress TNF production. In addition, the activity of *L. reuteri* CM and CP washes was partially blocked by H_2_ receptor antagonists, suggesting the presence of a second factor and/or pathway. The loss of TNF-inhibitory activity was comparable between the mutants and treatment with specific H_2_ receptor antagonists. In comparison, the activity of the B3 fraction, which contained histamine, tryptophan and phenylalanine, was completely blocked by H_2_ receptor antagonists, suggesting that histamine was the only active immunomodulin present in that fraction. Mouse studies are being performed to understand the importance of histamine as an immunomodulatory factor *in vivo*. In the future, it would be ideal to perform *in vivo* studies in which immunomodulins could be selectively added and removed via targeted mutagenesis to determine the importance and mechanism of action of each secretory factor produced by probiotic strains.

In addition to the role of histamine in immunomodulation, histamine is a known neurotransmitter [63]. Production of histamine by *L. reuteri* 6475 may influence signaling in the enteric nervous system, potentially impacting pain perception and gut motility. *In vivo* studies are needed to gain a greater understanding of the role of *L. reuteri*-produced histamine in the gut and its impact on gut health and disease. The production of microbial metabolites in the intestine and different mucosal surfaces may provide compounds involved in inter-kingdom signaling that promote mammalian health and immunologic homeostasis. Facilitated conversion of dietary components by bacterial species of the gut microbiome may provide an important bridge for enhancement of our understanding of the impact of diet on human health and disease.

## Materials and Methods

### Bacterial strains and culture conditions

All bacterial strains used in this study are described in Table 1. *L. reuteri* strains were cultured under anaerobic conditions for 16–18 h in deMan, Rogosa, Sharpe media (Difco, Franklin Lakes, NJ), and inoculated into semi-defined media, LDMIII (OD_600_ adjusted to 0.1), which has been described previously [64]. The carbon source was either glucose (LDMIIIG) or sucrose (LDMIIIS). Each culture was grown for 24 h at 37°C in an anaerobic workstation (MACS MG-500, Microbiology International, Frederick, MD) supplied with a mixture of 10% CO_2_, 10% H_2_, and 80% N_2_. At stationary phase (24 h), the cells were pelleted (4000×*g*, 10 min). Cell pellets and bacteria cell-free supernatants were further processed for HPLC separation and testing in a TNF inhibition bioassay.

**Table 1:** Bacterial strains and probes used in this study.

Bacterial Strains	Description*	Source
*L. reuteri* ATCC PTA 6475	Isolate from Finnish mother's milk	BioGaia AB (Stockholm, Sweden)
RPRB0000	*L. reuteri* expressing RecT	[33]
RPRB3002 – *rpoB* (HMPREF0536_0828)	*L. reuteri*::*oJP577* (RpoB: T487S, H488R); Rif^R^	[33]
RPRB3004 – *hdcP* (HMPREF0536_1229)	*L. reuteri*::*oJP577*::*oJP738* (RpoB: T487S, H488R; 1229: P25G, Q26X); Rif^R^	This study
RPRB3005 – *hdcA* (HMPREF0536_1230)	*L. reuteri*::*oJP577*::*oJP741* (RpoB: T487S, H488R; 1230: S83X, F84K); Rif^R^	This study
RPRB3006 – *hdcB* (HMPREF0536_1231)	*L. reuteri*::*oJP577*::*o1231* (RpoB: T487S, H488R; 1231: K31A, L32X); Rif^R^	This study

Primer Name	Primer Sequence	Universal Probe Number
rpoB-F	cgtgatacttcattacgtgttcct	77
rpoB-R	agtgaagactttaacatcttggatga	
hdcA-F	gcactaacgataaccgtcgtc	1
hdcA-R	cacccttattagcacaaacaatga	
hdcB-F	tgcaagatcggatatcacaaa	162
hdcB-R	ttgacgaattcaaccttctttaca	
hdcP-F	tccctacggataccaagcac	15
hdcP-R	agaggaacgctaagacaccaat	

*Rif^R^: rifampicin-resistant; RpoB: DNA-directed RNA polymerase (locus tag HMPREF0536_0828). Resultant amino acid changes after incorporation of the oligonucleotide are listed after each gene, and X represents a stop codon.

### Cell line and reagents

In vitro experiments were performed with THP-1 cells (human monocytoid cell line, ATCC number TIB-202, ATCC, Manassas, VA) maintained in RPMI (ATCC) and heat-inactivated fetal bovine serum (Invitrogen, Carlsbad, CA) at 37°C, 5% CO_2_. MEK1/2, phospho-MEK1/2, ERK1/2, and phospho-ERK1/2 antibodies and MEK inhibitor U0126 were from Cell Signaling Technology (Danvers, MA), and the β-Actin, H_1_R and H_2_R antibodies were from Abcam (Cambridge, MA). Ammonium acetate, glacial acetic acid and sodium hydroxide were from Mallinckrodt Baker (formerly J.T. Baker, Phillipsburg, NJ, USA). HPLC-grade water, methanol, and chloroform were from Fisher Scientific (Pittsburg, PA). Formic acid and histamine dihydrochloride were from Acros Organics (Geel, Belgium). Histamine-α,α,β,β-d_4_ dihydrochloride was from C/D/N Isotopes Inc. (Pointe-Claire, Quebec, Canada). All other reagents were obtained from Sigma (St. Louis, MO) unless otherwise stated.

### HILIC-HPLC separation of cell wall associated factors

Cell pellets from strain 6475 grown in either LDMIIIG or LDMIIIS were washed with ice cold 0.1% TFA. The cell suspension was centrifuged for 10 min, 4000×*g* at 4°C. Supernatants were filtered through polyvinylidene fluoride (PVDF) membrane filters (0.45 µm pore size, Millipore, Bedford, MA), lyophilized, and resuspended in 0.1% formic acid. The resuspended sample was size-fractionated with Amicon Ultra-15 centrifugal filter units using ultracel-3 membrane (Millipore). The filtrate was dried down and dissolved with 100% acetonitrile before fractionation through a PolyLC Hydroxyethyl column with a gradient of 100–0% acetonitrile, 0.1% formic acid. The sample was run for 25 min and 25 fractions (A1–C1) were collected at 10 mL/min/tube. Each fraction was lyophilized, resuspended in 0.1% acetic acid, and lyophilized again for testing in a TNF inhibition bioassay.

### TNF inhibition bioassay and TNF ELISA

Bacterial supernatants from 24 h LDMIII cultures were filter-sterilized using PVDF membrane filters (0.22 µm pore size, Millipore) and size fractionated as described above. The filtrate (<3 kDa) was dried and resuspended in RPMI medium to make conditioned media (CM). All CM was normalized by volume to an OD_600_ = 1.5. Lyophilized fractions from the HILIC-HPLC separation were resuspended in 10 mg/mL ammonium bicarbonate, dried, and resuspended in RPMI. CM and TFA-treated cell pellet fractions were tested for their ability to modulate TNF production in monocytoid cells. THP-1 cells (5×10^4^ cells) were stimulated to produce TNF by the addition of 100 ng/mL Pam_3_Cys-SKKKK (EMC Microcollections, Tuebingen, Germany). Inhibitors – ranitidine or cimetidine (1–100 nM), chlorpheniramine or pyrilamine (100 nM), U0126 (10 µM), and H89 (10 nM) – were added to the THP-1 cells followed by *L. reuteri* CM or HILIC-HPLC fractions (5% v/v), histamine (10 nM), or dibutyryl cAMP (0.1–1000 nM). Plates were incubated at 37°C and 5% CO_2_ for 3.5 h. Quantitative ELISAs were used to determine TNF according to the manufacturer's instructions (R&D Systems, Minneapolis, MN).

### NMR analysis of select HILIC-HPLC fractions

Protocols have been developed for characterizing metabolites using either 1D ^1^H NMR [65] or 2D HSQC and TOCSY [27]. The NMR samples were prepared by adding D_2_O (D, 99.9%, Cambridge Isotope Laboratories) and DSS (Sigma-Aldrich) to the lyophilized HILIC-HPLC fractions. The 1D ^1^H, 2D TOCSY, and natural abundance ^1^H-^13^C HSQC experiments were carried out at 25°C on Bruker Avance 500 MHz instrument equipped with triple-resonance z-gradient 5 mm probe. The spectral width, number of Free Induction Decays and the acquisition points for the 1D ^1^H experiments were 10000 Hz, 512 and 64 k, respectively. The TOCSY [66] experiment with 3-9-19 WATERGATE [67] was performed with 2048×256 data points in direct (with 160 number of scans) and in-direct dimensions, respectively. The mixing time was 70 ms with DIPSI-2 sequence [68]. The spectral widths of direct and in-direct dimensions were 8012 Hz and 5497 Hz, respectively. The ^1^H-^13^C HSQC experiment [69] with gradient coherence selection [70] was acquired with 2048×100 data points in ^1^H (with 1440 number of scans) and ^13^C dimensions, respectively. The spectral widths of ^1^H and ^13^C dimensions were 8012 Hz and 6788 Hz, respectively. The 2D data were processed with NMRPipe [71] and analyzed with NMRView [72] and MetaboMiner [27] softwares. The ^13^C time domain of HSQC was doubled using linear prediction [73] before Fourier transformation.

### Mass spectrometry analysis and quantification of histamine

HILIC-HPLC fractions were analyzed by ESI-qTOF MS on an Agilent 6520 mass spectrometer (Agilent, Santa Clara, CA) equipped with a 1260 NanoPump and a chip cube ESI source with a chip for direct sample infusion. To remove interfering TFA, HILIC-HPLC fractions were lyophilized and resuspended in 0.1% formic acid. The samples were analyzed in MS and MS/MS. Culture supernatants were lyophilized and resuspended in HPLC-Water. The samples were adjusted to pH 11 using sodium hydroxide solution (5 M) and equal volumes of chloroform were added and vigorously shaken for liquid-liquid extraction. The organic phase was collected, dried and resuspended in 0.1% formic acid. Quantification of histamine in both types of samples (HILIC fractions and culture supernatants) was performed on an ESI triple quadrupole (QQQ) mass spectrometer, Agilent 6410 Series equipped with an Agilent 1100 HPLC system and a Zorbax SB-C18 HPLC column (150 mm×0.5 mm, 5 µm particles, Agilent). Samples were spiked with deuterated histamine (histamine-α,α,β,β-d_4_ dihydrochloride) as an internal standard (10 pg/µL) in ammonium acetate (15 mM, pH 3.5). Samples were injected into the HPLC system and then eluted from the column using a short gradient of 15 mM ammonium acetate (pH 3.5, solvent A) and methanol (100%, solvent B). The elution profile for solvent B was: 0.0–5.0 min, 10%; 5.0–5.1 min, 10–40%; and 5.1–8.0 min, 40%. For QQQ-MS quantification of histamine [74], the following fragmentation transitions were used in multiple-reaction monitoring mode: m/z 112.1 to m/z 95.1 for quantification; the fragment at m/z 68.1 served as a qualifier. For the deuterated standard the transitions m/z 116.1 to m/z 99.1 were used as quantifier and the fragment at m/z 72.1 served as a qualifier. The ESI probe tip was at 3.5 kV, nitrogen was used as nebulizer at 300°C at 15 psi, the insource fragmentor voltage was 105 V, and the potentials across the collision cell were 12 V and 24 V for quantifier and qualifier ions, respectively.

### Quantification of histamine by ELISA

Wild-type *L. reuteri* 6475, *hdcA*, *hdcB* and *hdcP* mutants were grown as described above in LDMIIIG supplemented with 4 mg/mL L-histidine. Cultures were harvested at 24 h, centrifuged (1500×*g*), and filter-sterilized with 0.22-µm PVDF filters. Samples were diluted in PBS, and histamine concentrations were determined using the Histamine ELISA kit (Neogen, Lexington, KY) according to the manufacturer's instructions. Absorbance was measured with a Spectramax 340PC, and data were analyzed using GraphPad Prism 5 software. Data were corrected with values obtained from the background control (LDMIIIG+1% MRS) and normalized to the same OD_600_ of 24 h LDMIIIG cultures.

### Transcriptomic comparison of *L. reuteri* 6475 grown with different carbon sources

*L. reuteri* 6475 was cultured in LDMIIIS or LDMIIIG to stationary phase. For expression analyses, three biological replicates were performed with dye-swap experiments for each comparison. Following mRNA isolation, cDNA synthesis, labeling, and hybridization were performed as previously described [75], [76]. Information regarding the microarray platforms can be found at the NCBI Gene Expression Omnibus (GEO; http://www.ncbi.nlm.nih.gov/geo/) under GEO platform GPL7541. The complete set of microarray data can be found under the GEO series accession GSE32612. Microarray data analysis was performed as previously described [75].

### Construction of *L. reuteri* histidine decarboxylase mutants

Mutants were generated using RecT-mediated oligonucleotide recombineering as previously described [32], [33]. *L. reuteri* expressing RecT (strain RPRB0000) was used to construct mutations in *rpoB* (locus tag HMPREF0536_0828 (ZP_03961568)) and the target genes located in the histidine decarboxylase gene cluster HMPREF0536_1229 (ZP_03961969), HMPREF0536_1230 (ZP_03961970) and HMPREF0536_1231 (ZP_03961971) to yield strains RPRB3002, RPRB3004, RPRB3005 and RPRB3006, respectively. Mutations were verified by PCR, and the integrity was confirmed by sequence analysis. Oligonucleotides for recombineering and screening purposes are available upon request.

### Expression studies of the *L. reuteri* histidine decarboxylase gene cluster

*L. reuteri* 6475 was grown as described above in LDMIIIG with or without 4 mg/mL L-histidine. At 16 h post-inoculation, the OD_600_ was measured, and an equal volume of ice-cold methanol was added to each culture. Half of each culture-methanol mixture was pelleted by centrifugation (1500×*g*), resuspended in STE buffer (6.7% sucrose, 50 mM Tris [pH 8.0], 1 mM EDTA) with RNaseOUT (Invitrogen, Carlsbad, CA) and mechanically disrupted using a Fastprep-24 Sample Preparation System (M.P. Biomedicals, Irvine, CA) with 0.1 mm glass beads at 4.0 m/s for 20 s. Each sample was centrifuged briefly, RTL Plus buffer (Qiagen, Valencia, CA) was added and mechanical disruption was repeated. RNA extraction was performed with the AllPrep RNA/DNA/Protein Mini-kit with on-column DNase I treatment (Qiagen). DNA was removed using TURBO DNA-free Kit (Applied Biosystems, Foster City, CA). cDNA synthesis with Superscript III Reverse Transcriptase (Invitrogen) was performed using 3 µg RNA.

Expression of the hdc genes was analyzed using quantitative real-time PCR. All primers were designed using the Universal ProbeLibrary Assay Design Center (Roche Applied Science, Indianapolis, IN) and are described in Table 1. The RNA polymerase β-subunit (*rpoB*) gene was used as a reference gene. PCR reactions were set up using 2× FastStart Universal Probe Master (Rox) (Roche Applied Science) and the cDNA described above, with final concentrations of 200 nM for each primer and 100 nM for each probe. All PCR reactions were performed using the ViiA 7 Real-Time PCR System. Cycling parameters were as follows: program 1; 1 cycle of 25°C for 2 min, 1 cycle of 95°C for 10 min, program 2; 50 three-step cycles of 95°C for 15 s, 60°C for 1 min and 72°C for 1 min, program 3; hold at 4°C. Fluorescence was detected after the extension step in each cycle. The 2^−ΔΔCT^ method was used to calculate relative changes in *hdc* gene expression when LDMIIIG was supplemented with L-histidine.

### Quantification of cAMP by ELISA

Intracellular levels of cAMP in THP-1 cell lysates were determined using a quantitative ELISA (R&D Systems). THP-1 cells were stimulated with PCK in the presence of medium control, *L. reuteri* 6475, and histamine with and without ranitidine (as described for the TNF inhibition bioassay) for 10 min. Cell pellets were collected by centrifugation (3000×*g*, 5 min, 4°C) and resuspended in lysis buffer 5 (included in the cAMP ELISA kit). Cells were lysed by two freeze/thaw cycles and centrifuged at 600×*g*, 10 min, 4°C to remove cellular debris. Quantification of cAMP was performed by competitive binding ELISA according to the manufacturer's instructions.

### MEK1/2 and ERK1/2 detection by immunoblot

THP-1 cells were lysed in ice-cold lysis buffer consisting of 50 mM Tris, pH 7.4, 250 mM NaCl, 5 mM EDTA, 50 mM NaF, 1 mM Na_3_VO_4_, 1% v/v Nonidet P40, 0.2% v/v NaN_3_, and protease and phosphatase inhibitors. Lysates were incubated on ice for 30 min, vortexed every 10 min, and cleared by centrifugation at 13,000×*g* for 10 min at 4°C. Protein concentrations were measured using the Quant-iT™ Protein Assay kit (Invitrogen) and a Qubit fluorometer according to the manufacturer's instructions. Equal amounts of proteins were loaded onto electrophoresis gels. Analysis of ERK1/2 activation was performed using specific phospho-ERK1/2 antibodies. Cell extracts were loaded in a 10% SDS-polyacrylamide gel and transferred to PVDF membranes (Bio-Rad, Hercules, CA). Membranes were blocked overnight at 4°C in blocking buffer (Li-Cor Biosciences, Lincoln, NE). After several washes, membranes were probed with ERK1/2, phospho-ERK1/2 or β-Actin specific antibodies diluted in blocking buffer (Li-Cor) for 1 h at room temperature. After washes, membranes were incubated with the appropriate horseradish peroxidase-conjugated secondary antibody for 1 h at room temperature, and blots were developed using chemiluminescent detection. Analysis of MEK1/2 activation was performed as described above except primary phospho-MEK1/2 and MEK1/2 antibody incubation was 17 hrs at 4°C. Pixel density analysis was performed in a FluorChem HD2 system (Alpha Innotech, Santa Clara, CA).

## Supporting Information

Figure S1 **TNF-inhibitory compounds were isolated in three distinct HILIC-HPLC fractions.** Compounds from TFA-treated *L. reuteri* cell pellets were separated based on relative hydrophobicity. **A.** TNF-inhibitory compounds from *L. reuteri* 6475 grown in a glucose-containing medium were isolated in 3 fractions (B3, B5, and B6). Results represent the mean ± SD (n = 3), ^*^p value<0.05. **B.** No TNF-inhibitory compounds from *L. reuteri* 6475 grown in a sucrose-containing medium were isolated by HILIC-HPLC.
(TIF)

Figure S2 **Triple quadrupole mass spectrometry standard curve.** Standard curve generated from deuterated histamine and used for the triple quadrupole MS quantification of histamine in HILIC-HPLC fractions and bacterial culture supernatant. Each sample was spiked with deuterated histamine as an internal standard.
(TIF)

Figure S3 **Genes in the histidine decarboxylase gene cluster were necessary for production of histamine.** **A.** Quantitative real-time PCR demonstrated increased expression of all three hdc genes, *hdcA*, *hdcB*, and *hdcP*, when *L. reuteri* 6475 was grown in LDMIIIG medium supplemented with 4 mg/mL L-histidine compared to unsupplemented LDMIIIG. Gene expression data were normalized using *rpoB* as a reference gene. Expression ratios of each gene (histidine-supplemented versus unsupplemented LDMIIIG) were calculated. Results represent the mean ± SD (n = 3), ^**^p value<0.005, ^*^p value<0.05 compared to the theoretical mean of 1.0. **B.** Quantification of secreted *L. reuteri*-derived histamine by a histamine-specific ELISA demonstrated decreased histamine production in all three hdc gene mutants, *hdcA*, *hdcB*, and *hdcP*, compared to wild-type *L. reuteri* 6475. Results represent the mean ± SD (n = 3), ^***^p value<0.001 compared to wild-type 6475.
(TIF)

Figure S4 **THP-1 cells express the H_1_ and H_2_ receptors.** Unstimulated THP-1 cells were examined for cell surface expression of the histamine H_1_ and H_2_ receptors. Cells were labeled with rabbit anti-human H_1_R or H_2_R pAb and FITC-conjugated goat anti-rabbit IgG pAb or FITC-conjugated goat anti-rabbit IgG pAb alone (IgG Control) and analyzed with FACS. Shown is one representative experiment of at least three.
(TIF)

Figure S5 **Elevated cAMP inhibited TNF production from activated human monocytoid cells.** Treatment of TLR2-stimulated THP-1 cells with a synthetic analog of cAMP, dcAMP, was sufficient to inhibit TNF production. Results represent the mean ± SD (n = 3), ^*^p value<0.05 compared to medium control.
(TIF)

Figure S6 **Pixel density analysis of MEK1/2 and ERK1/2 immunoblots.** Immunoblots of MEK1/2 and ERK1/2 were quantified by pixel density analysis. **A.** MEK1/2. Results represent the mean ± SEM (n = 3), ^*^p value<0.05 compared to medium control. **B.** ERK1/2. Results represent the mean ± SEM (n = 3), ^*^p value<0.05 compared to medium control, ^#^p value<0.05 compared to *L. reuteri* 6475, ^##^p value<0.05 compared to histamine.
(TIF)

Figure S7 **Inhibition of ERK1/2 activation suppressed TNF production from activated human monocytoid cells.** Inhibition of the MEK/ERK signaling pathway with a MEK-specific inhibitor, U0126, was sufficient to block TNF production. Results represent the mean ± SD (n = 3), ^*^p value<0.05 compared to RPMI medium control, ^#^p value<0.05 compared to LDMIIIG medium control.
(TIF)

Figure S8 **TNF-inhibitory phenotype was modified by the carbon source in the growth medium.** Supplementing the growth medium with various simple sugars, such as glucose, glucose+fructose, and sucrose, altered the ability of TFA-treated cell pellets (CP) from *L. reuteri* 6475 to inhibit TNF production. Results represent the mean ± SD (n = 3), ^*^p value<0.05 compared to medium control, ^#^p value<0.05 compared to *L. reuteri* 6475 CP (glucose).
(TIF)

Figure S9 **A complete histidine decarboxylase gene cluster was found only in lactobacilli.** Analysis of the HMP reference genomes (GI bacteria) for histidine decarboxylase gene homologs revealed that out of 349 reference genomes, only four bacterial strains contained the complete histidine decarboxylase gene cluster. The strains were *L. reuteri* JCM 112, *L. reuteri* MM2-3, *L. reuteri* 6475, and *L. vaginalis* ATCC 49540.
(TIF)
