# Supplementary figures and images for: Histamine Derived from Probiotic Lactobacillus reuteri Suppresses TNF via Modulation of PKA and ERK Signaling
Source: PLoS One. 2012 Feb 22;7(2):e31951. doi: 10.1371/journal.pone.0031951 (PMC3285189; doi:10.1371/journal.pone.0031951)

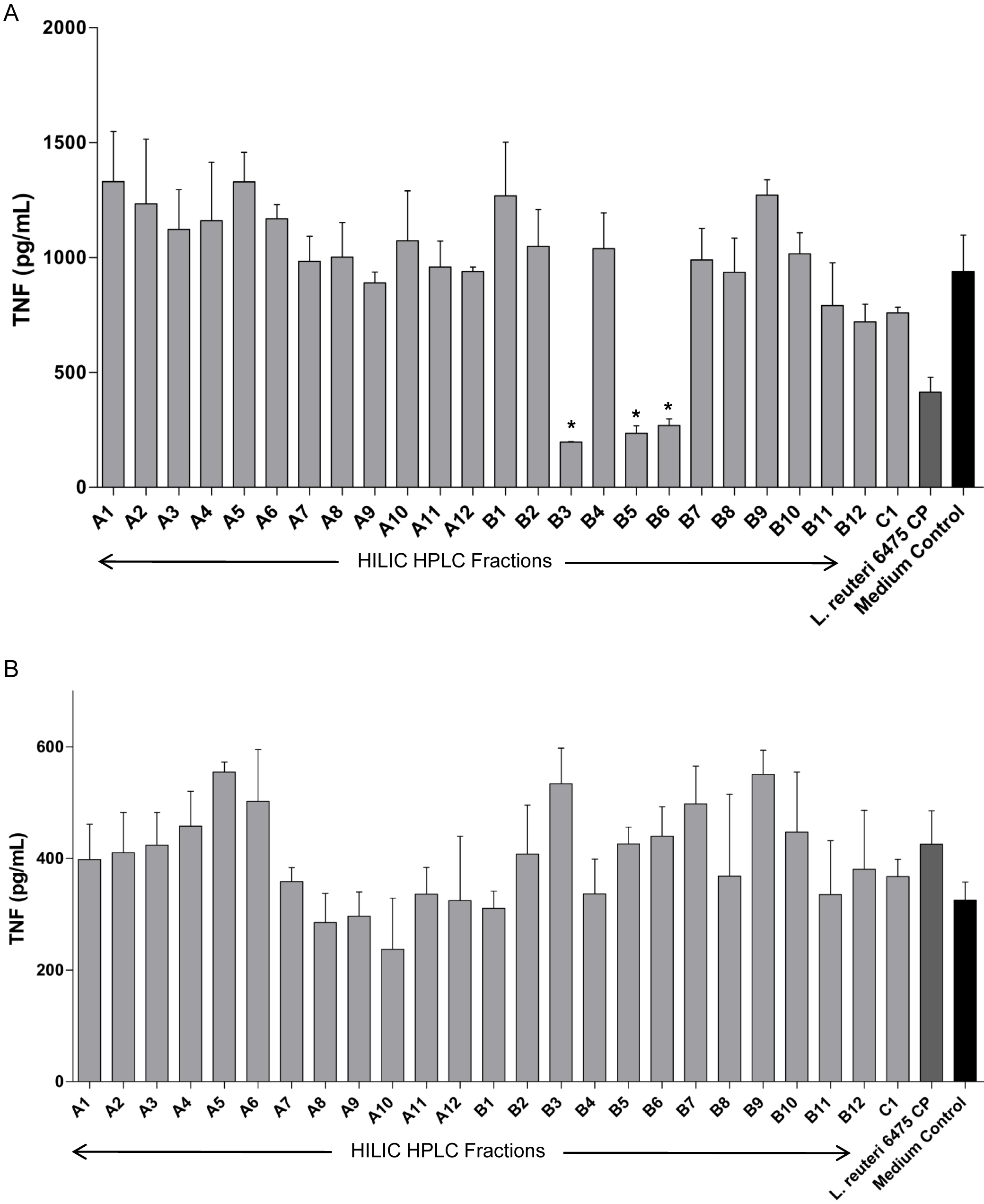

Supplement: Figure S1 — TNF-inhibitory compounds were isolated in three distinct HILIC-HPLC fractions. Compounds from TFA-treated L. reuteri cell pellets were separated based on relative hydrophobicity. A. TNF-inhibitory compounds from L. reuteri 6475 grown in a glucose-containing medium were isolated in 3 fractions (B3, B5, and B6). Results represent the mean ± SD (n = 3), *p value<0.05. B. No TNF-inhibitory compounds from L. reuteri 6475 grown in a sucrose-containing medium were isolated by HILIC-HPLC. (TIF) [file pone.0031951.s001.tif]

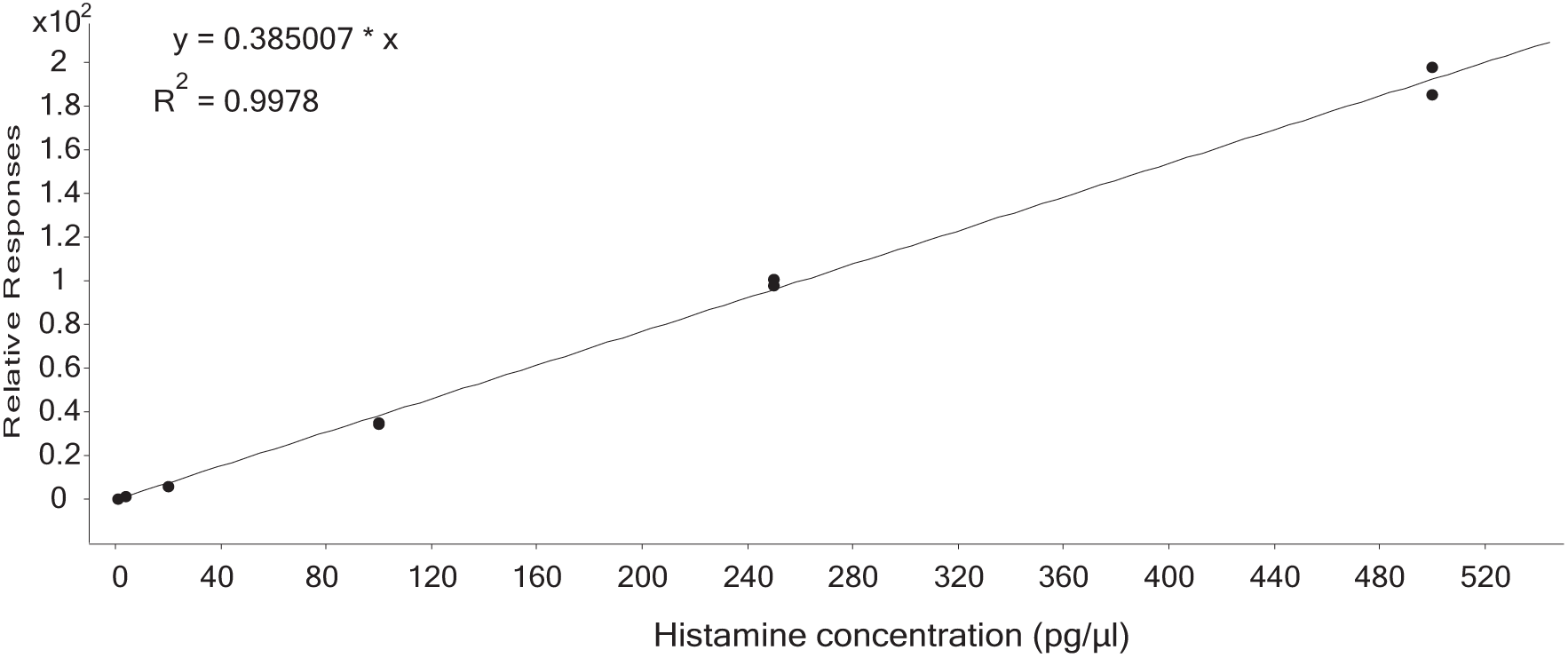

Supplement: Figure S2 — Triple quadrupole mass spectrometry standard curve. Standard curve generated from deuterated histamine and used for the triple quadrupole MS quantification of histamine in HILIC-HPLC fractions and bacterial culture supernatant. Each sample was spiked with deuterated histamine as an internal standard. (TIF) [file pone.0031951.s002.tif]

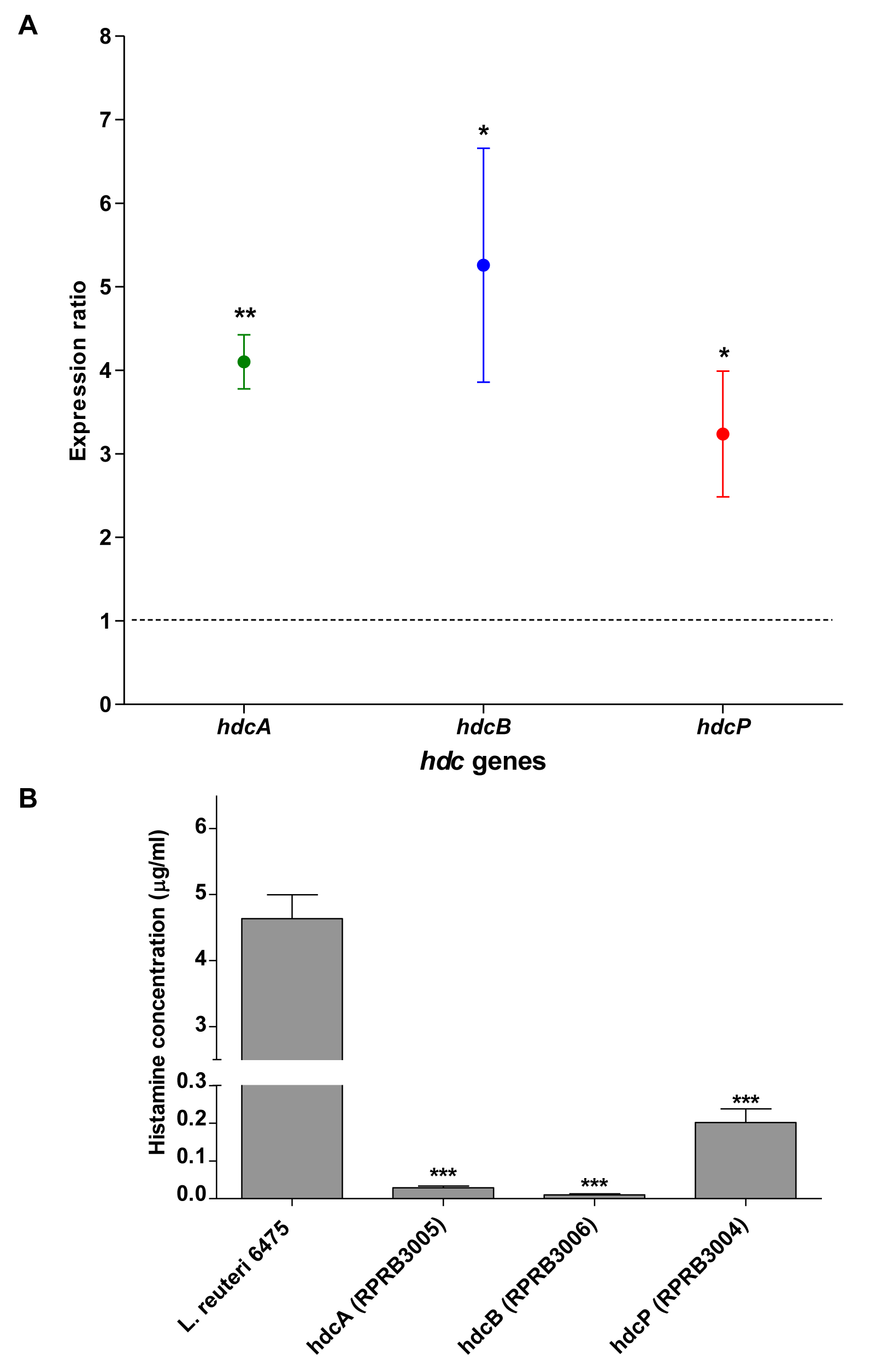

Supplement: Figure S3 — Genes in the histidine decarboxylase gene cluster were necessary for production of histamine. A. Quantitative real-time PCR demonstrated increased expression of all three hdc genes, hdcA, hdcB, and hdcP, when L. reuteri 6475 was grown in LDMIIIG medium supplemented with 4 mg/mL L-histidine compared to unsupplemented LDMIIIG. Gene expression data were normalized using rpoB as a reference gene. Expression ratios of each gene (histidine-supplemented versus unsupplemented LDMIIIG) were calculated. Results represent the mean ± SD (n = 3), **p value<0.005, *p value<0.05 compared to the theoretical mean of 1.0. B. Quantification of secreted L. reuteri-derived histamine by a histamine-specific ELISA demonstrated decreased histamine production in all three hdc gene mutants, hdcA, hdcB, and hdcP, compared to wild-type L. reuteri 6475. Results represent the mean ± SD (n = 3), ***p value<0.001 compared to wild-type 6475. (TIF) [file pone.0031951.s003.tif]

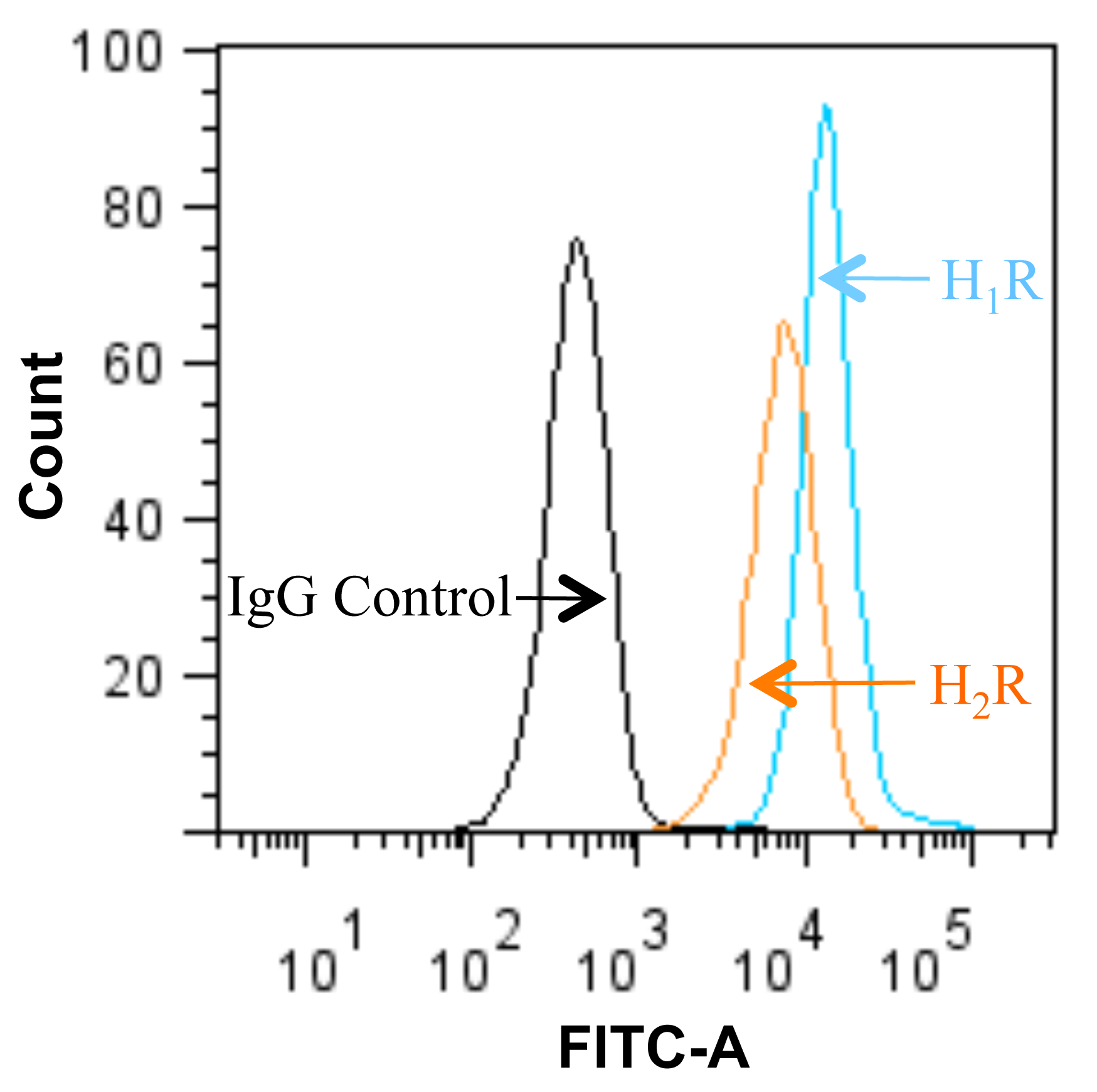

Supplement: Figure S4 — THP-1 cells express the H1 and H2 receptors. Unstimulated THP-1 cells were examined for cell surface expression of the histamine H1 and H2 receptors. Cells were labeled with rabbit anti-human H1R or H2R pAb and FITC-conjugated goat anti-rabbit IgG pAb or FITC-conjugated goat anti-rabbit IgG pAb alone (IgG Control) and analyzed with FACS. Shown is one representative experiment of at least three. (TIF) [file pone.0031951.s004.tif]

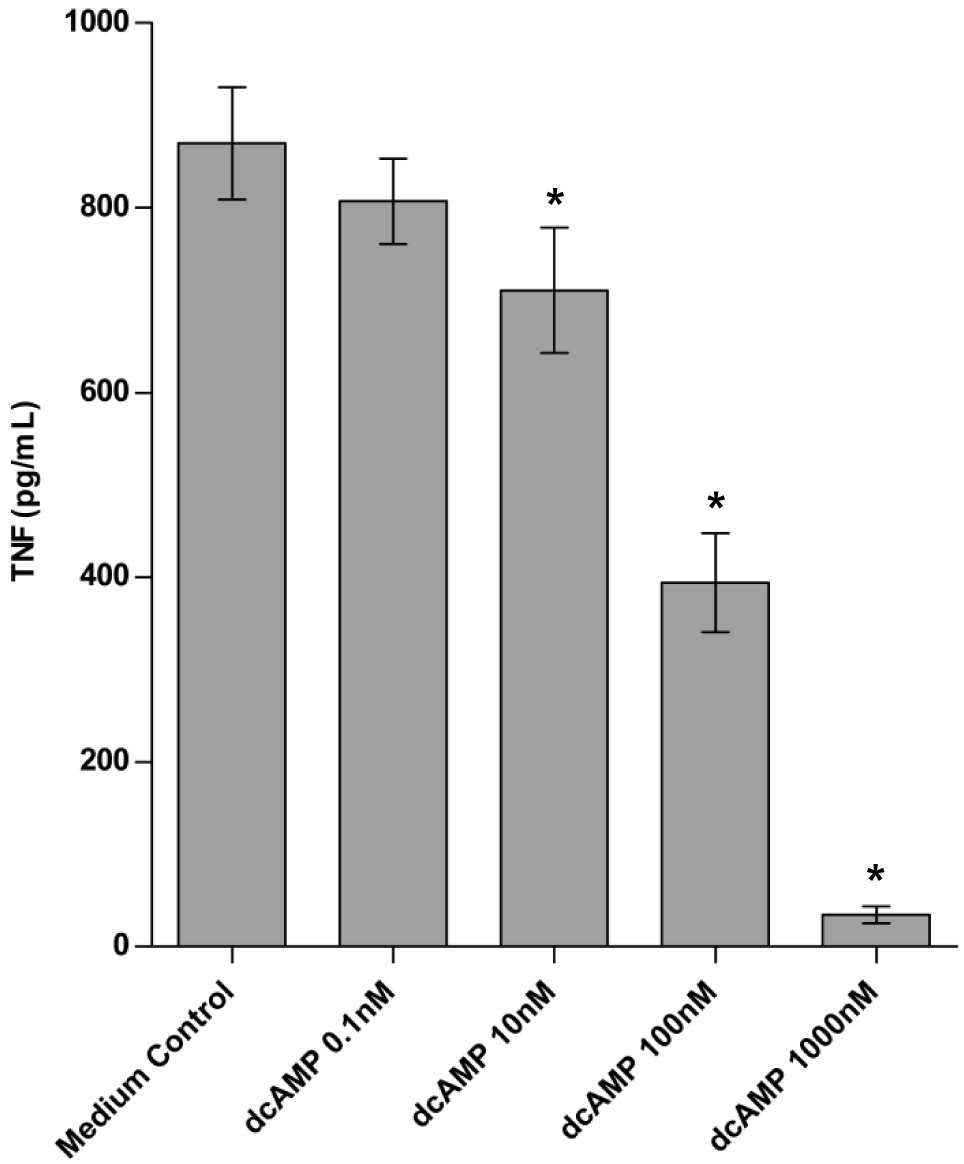

Supplement: Figure S5 — Elevated cAMP inhibited TNF production from activated human monocytoid cells. Treatment of TLR2-stimulated THP-1 cells with a synthetic analog of cAMP, dcAMP, was sufficient to inhibit TNF production. Results represent the mean ± SD (n = 3), *p value<0.05 compared to medium control. (TIF) [file pone.0031951.s005.tif]

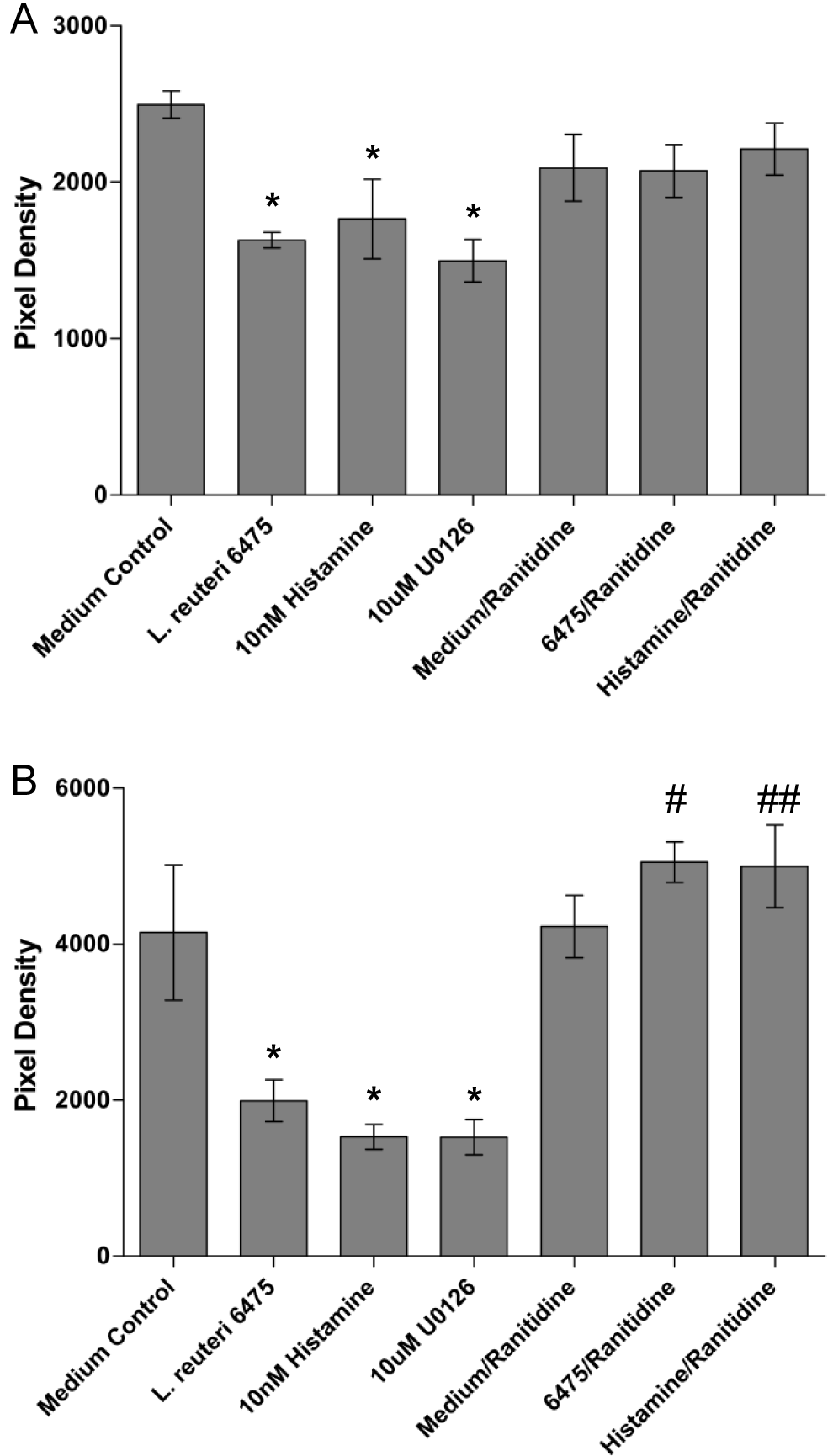

Supplement: Figure S6 — Pixel density analysis of MEK1/2 and ERK1/2 immunoblots. Immunoblots of MEK1/2 and ERK1/2 were quantified by pixel density analysis. A. MEK1/2. Results represent the mean ± SEM (n = 3), *p value<0.05 compared to medium control. B. ERK1/2. Results represent the mean ± SEM (n = 3), *p value<0.05 compared to medium control, #p value<0.05 compared to L. reuteri 6475, ##p value<0.05 compared to histamine. (TIF) [file pone.0031951.s006.tif]

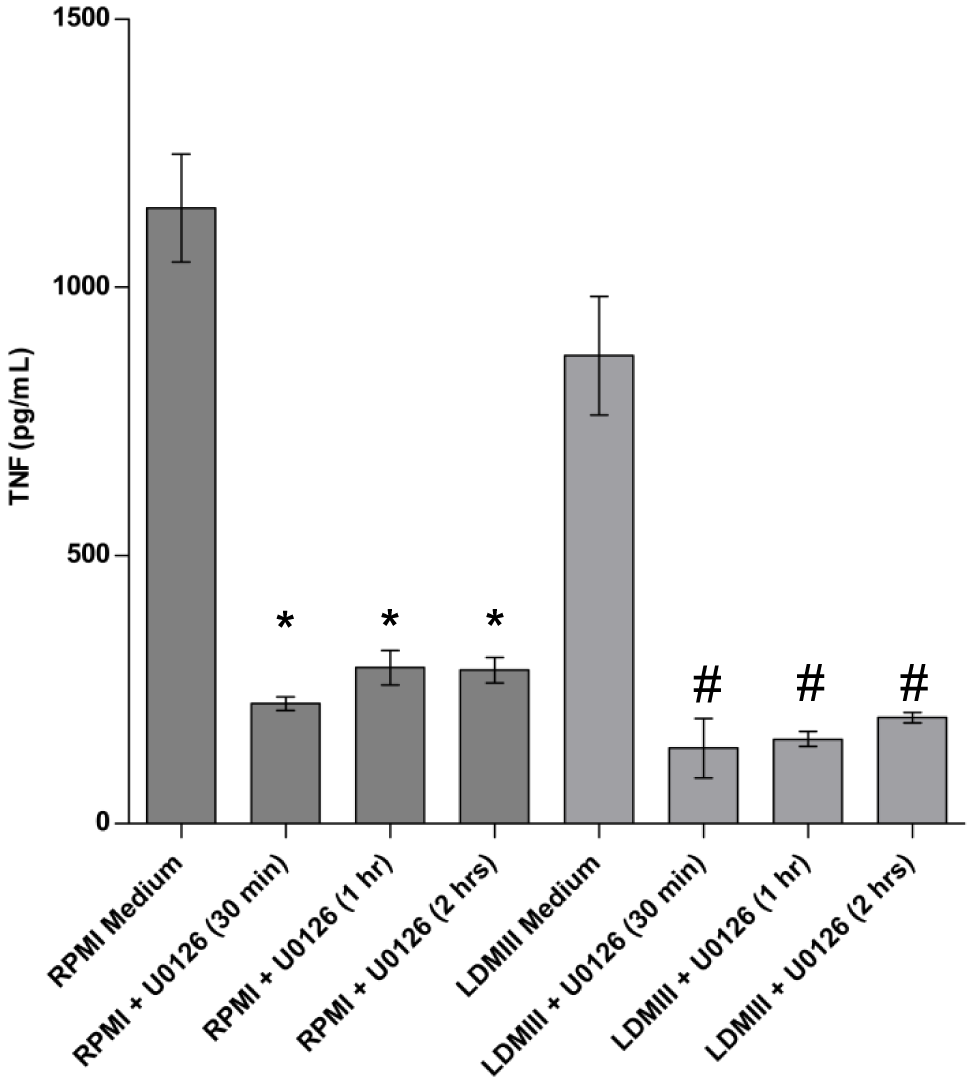

Supplement: Figure S7 — Inhibition of ERK1/2 activation suppressed TNF production from activated human monocytoid cells. Inhibition of the MEK/ERK signaling pathway with a MEK-specific inhibitor, U0126, was sufficient to block TNF production. Results represent the mean ± SD (n = 3), *p value<0.05 compared to RPMI medium control, #p value<0.05 compared to LDMIIIG medium control. (TIF) [file pone.0031951.s007.tif]

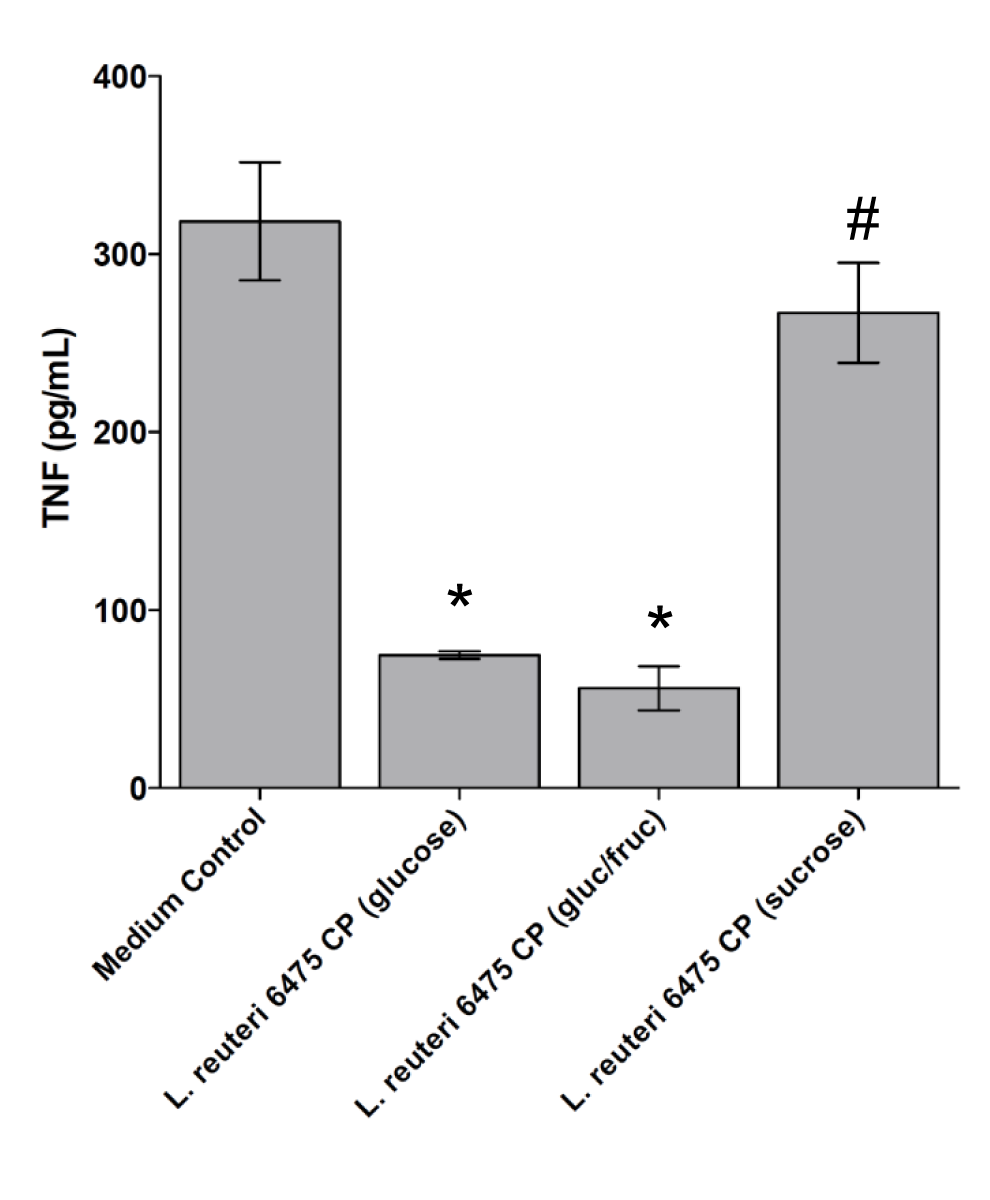

Supplement: Figure S8 — TNF-inhibitory phenotype was modified by the carbon source in the growth medium. Supplementing the growth medium with various simple sugars, such as glucose, glucose+fructose, and sucrose, altered the ability of TFA-treated cell pellets (CP) from L. reuteri 6475 to inhibit TNF production. Results represent the mean ± SD (n = 3), *p value<0.05 compared to medium control, #p value<0.05 compared to L. reuteri 6475 CP (glucose). (TIF) [file pone.0031951.s008.tif]

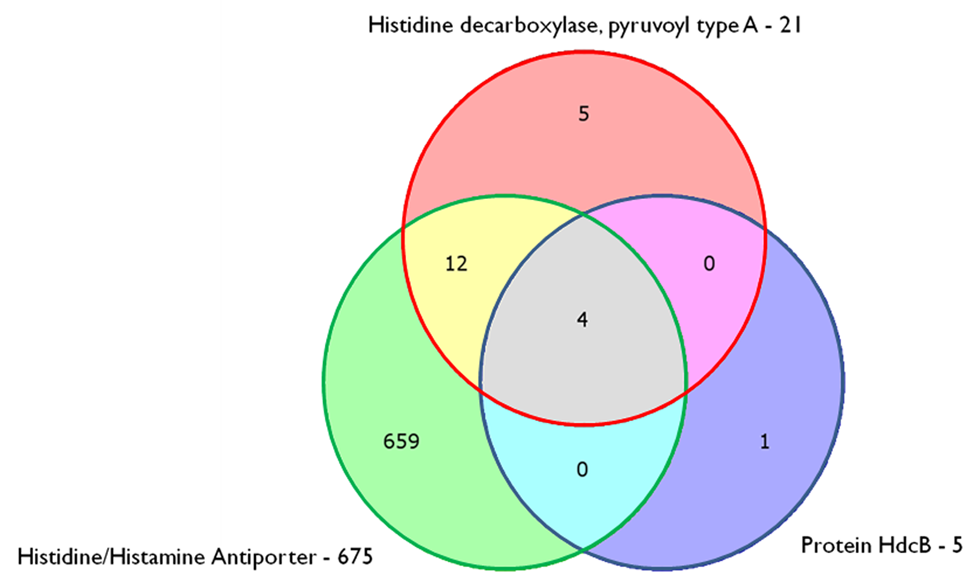

Supplement: Figure S9 — A complete histidine decarboxylase gene cluster was found only in lactobacilli. Analysis of the HMP reference genomes (GI bacteria) for histidine decarboxylase gene homologs revealed that out of 349 reference genomes, only four bacterial strains contained the complete histidine decarboxylase gene cluster. The strains were L. reuteri JCM 112, L. reuteri MM2-3, L. reuteri 6475, and L. vaginalis ATCC 49540. (TIF) [file pone.0031951.s009.tif]
